# Simple and efficient synthesis of bicyclic enol-carbamates: access to brabantamides and their analogues†

**DOI:** 10.1039/d0ra00796j

**Published:** 2020-02-13

**Authors:** Ondrej Záborský, Ľudmila Petrovičová, Jana Doháňošová, Ján Moncol, Róbert Fischer

**Affiliations:** Institute of Organic Chemistry, Catalysis and Petrochemistry, Slovak University of Technology in Bratislava Radlinského 9 Bratislava 81237 Slovak Republic ondrej.zaborsky@stuba.sk; Central Laboratories, Slovak University of Technology in Bratislava Radlinského 9 Bratislava 81237 Slovak Republic; Institute of Inorganic Chemistry, Technology and Materials, Slovak University of Technology in Bratislava Radlinského 9 Bratislava 81237 Slovak Republic

## Abstract

A novel synthetic approach towards the formation of the unusual bicyclic enol-carbamates, as found in brabantamides A–C, is reported. The bicyclic oxazolidinone framework was obtained in very good yield and with high *E*/*Z* selectivity from a readily available β-ketoester under mild reaction conditions using Tf_2_O and 2-chloropyridine tandem. The major *E* isomer was used in the synthesis of the brabantamide A analogue.

## Abstract 2

A simple and short synthesis of bicyclic enol-carbamates with high *E*/*Z* selectivity and the synthesis of brabantamide A analogue are presented.

## Graphical abstract

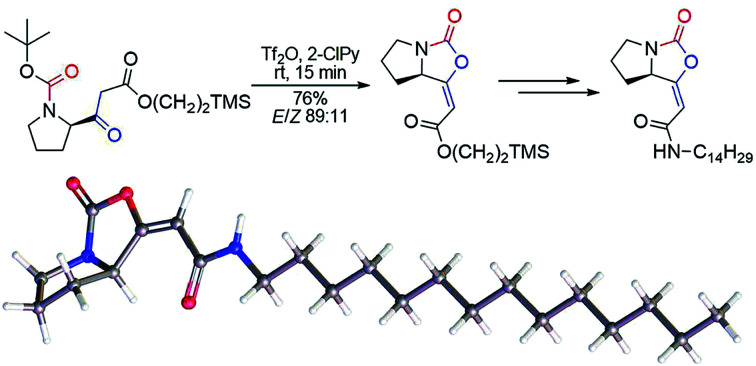

Brabantamides A–C (1–3) (Fig. 1) were first isolated in 2000 from the culture extracts of *Pseudomonas fluorescens*.^1^ They displayed nanomolar inhibitory activity towards lipoprotein-associated phospholipase A_2_ (Lp-PLA_2_) and therefore they could be used in the treatment of the inflammatory diseases such as atherosclerosis.^1–3^ It was found that the sugar moiety is not necessary for the biological activity against Lp-PLA_2_. In contrast, deglycosylated brabantamides A and C showed improved inhibitory activity compared to their natural counterparts.^3^ Moreover, simplified brabantamide analogues with amide functional group and long alkyl side chains showed even higher inhibitory effect.^4^ Brabantamides A–C also exhibit significant activity against Gram-positive bacteria, fungi, and oomycetes.^5,6^ A recent study confirmed that the bicyclic scaffold and the long lipophilic side chain are essential for the antibacterial activity.^7^

**Fig. 1:**
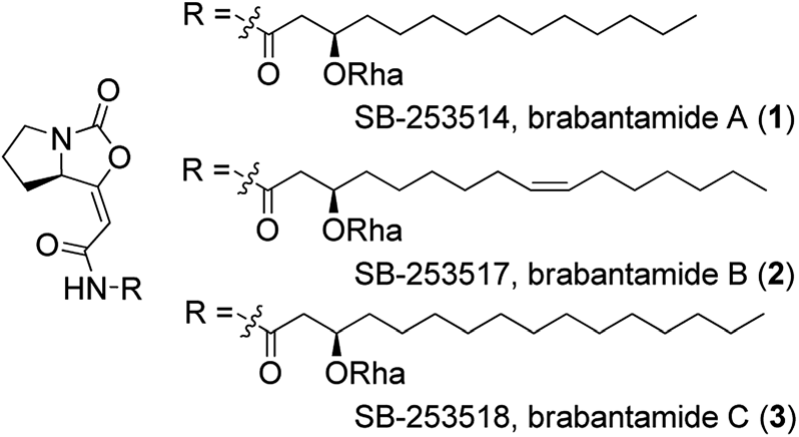
Structures of brabantamides A–C (1–3). Rha = rhamnose.

Surprisingly, only five reports concerning the synthesis of the bicyclic oxazolidinone with an exocyclic double bond have been reported to date.

In 2000, Pinto *et al.* prepared a series of analogues of brabantamide A where the enol-carbamate 5 was first obtained by direct iodocyclization from acetylene derivative 4 and then converted into key ester 6 by carbonylation of the corresponding vinyl iodide in the presence of 2-(trimethylsilyl)ethanol and PdCl_2_ (Scheme 1a).^4^ Another synthesis of the related esters 9 has been reported by Snider *et al.* in 2006, employing Wittig reaction between stabilized ylides and bicyclic oxazolidindione 8 synthesized from l-proline 7 (Scheme 1b).^8^ Shortly after, bicyclic enol-carbamate 10 with an exocyclic methylene group was synthesized in one step from acetylene derivative 4 using gold(i) catalysts (Scheme 1c).^9^ Very recently, Witte *et al.* reported the synthesis of series of *Z*-analogues of brabantamides 12 by cyclization of β-ketoamides 11 using CDI (Scheme 1d).^7^ As a part of our ongoing research program aimed at the utilization of trifluoromethanesulfonic anhydride (Tf_2_O)/2-halopyridine (2-XPy) tandem in the synthesis of bioactive natural products and their analogues, we envisioned that similar reaction conditions could be effectively used to form the bicyclic oxazolidinone framework as found in brabantamides.

**Scheme 1:**
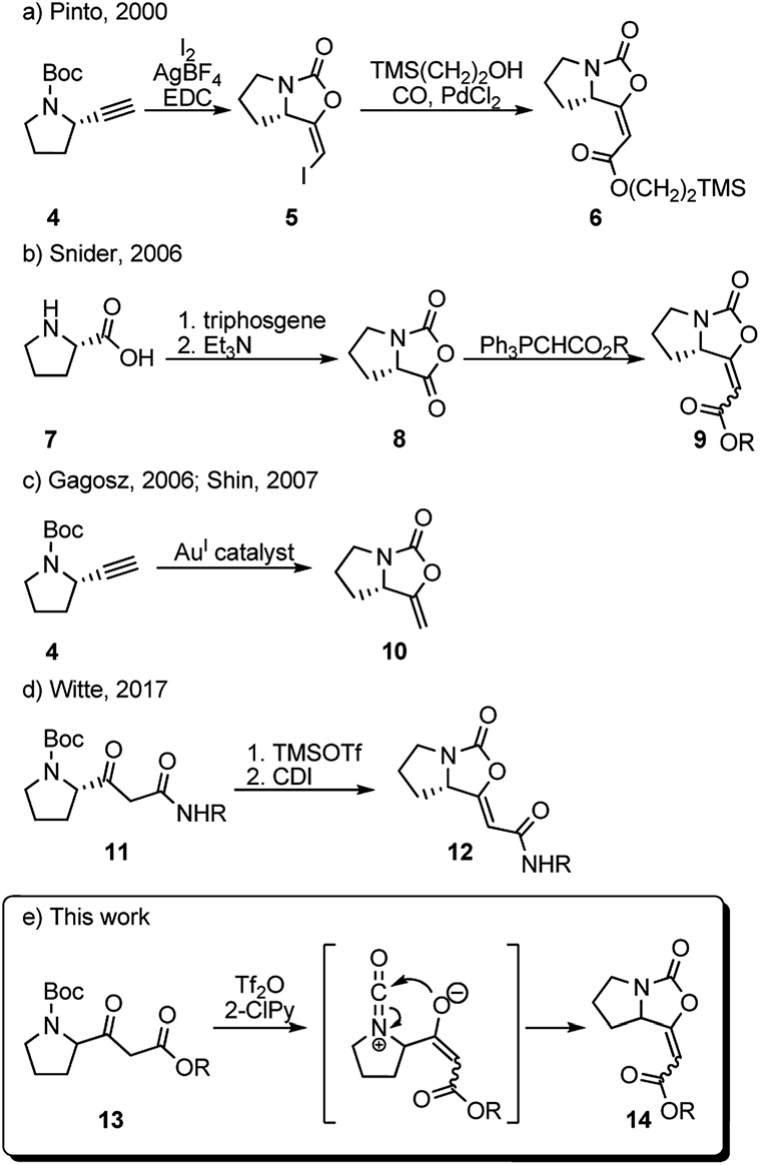
Literature syntheses of bicyclic enol-carbamates and method proposed herein.

The combination of Tf_2_O/2-XPy was extensively and successfully used in the amide activation^10,11^ as well as in generating isocyanate species from *N*-Boc and *N*-Cbz protected amines.^12^ We anticipated that the *N*-Boc-protected β-ketoester 13 could react in its enolate form with *in situ* generated isocyanate ion by intramolecular 5-*endo-dig* cyclization to give bicyclic enol-cyclocarbamate 14 (Scheme 1e).

At the start of our investigation, model β-ketoester 15, prepared from *N*-Boc-l-proline,^7^ was chosen as the model substrate to identify optimal reaction conditions (Table 1). According to Kokotos' protocol,^12*c*^ the initial using of 1.5 equivalents of Tf_2_O and 3 equivalents of 2-ClPy led to a full conversion of the substrate 15 in 15 minutes (monitored by TLC) (Table 1, entry 7). The inspection of ^1^H NMR spectrum of the crude reaction mixture confirmed the presence of only desired product 16 almost exclusively as *E* isomer (*E*/*Z* ratio 93 : 7) which was isolated in 53% combined yield. Gratifyingly, lowering the amount Tf_2_O (1.1 equiv.) resulted in a significant increase of the yield up to 80% with the slight decrease of *E* isomer 16a (Table 1, entry 8).^13^ Any variation of the amount of 2-ClPy did not have any positive impact on the reaction (Table 1, entries 9 and 10). The use of other 2-halopyridines reduced yield of 16 and prolonged reaction times were observed (Table 1, entries 11–13). For comparison, when we applied Witte's reaction conditions, yield dropped remarkably and *E*/*Z* selectivity disappeared completely (Table 1, entry 14). At last, we tested other bases commonly used in the combination with Tf_2_O. Triethylamine, 4-dimethylaminopyridine, pyridine, and 2,6-lutidine resulted only in traces of product 16 (Table 1, entries 2–5), as well as when no base was used (Table 1, entry 1). Using DBU, enol-carbamate 16 was formed in slightly improved *E*/*Z* ratio (Table 1, entry 6). Nevertheless, ^1^H NMR spectrum of the crude reaction mixture showed the formation of a large amount of unidentified by-products and desired product was isolated only in 41% yield.

**Table 1:** Optimization of the reaction conditions for cyclization of β-ketoester 15

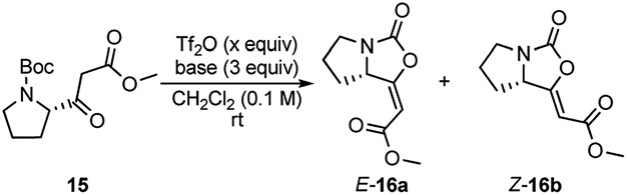					
Entry	Tf_2_O	Base	Time (min)	16a : 16b^a^	Yield^b^ (%)
1	1.5	—	60	—	–c
2	1.5	Et_3_N	60	—	–c
3	1.5	DMAP	60	—	–c
4	1.5	Pyridine	60	—	–c
5	1.5^d^	2,6-Lutidine	60	—	–c
6	1.5^d^	DBU	60	90 : 10	41^e^^,^^f^
7	1.5	2-ClPy	15	93 : 7	53^e^
**8**	**1.1**	**2-ClPy**	**15**	**85 : 15**	**80** e
9	1.1	2-ClPy (1.5 equiv.)	40	85 : 15	75^e^
10	1.1	2-ClPy (5 equiv.)	15	87 : 13	64^e^
11	1.1	2-FPy	15	89 : 11	71^e^
12	1.1	2-BrPy	70	86 : 14	73^e^
13	1.1	2-IPy	90	86 : 14	68^e^
14	Witte's protocol^g^		Overnight	50 : 50	36^e^

a Ratio determined by ^1^H NMR of the crude reaction mixture.

b Isolated combined yield.

c Traces of products.

d Reactions performed with 1.1 equiv. of Tf_2_O did not lead to full conversion of ester 15.

e Reactions were performed on 1 mmol of ester 15.

f Reaction mixture contained a large amount of unidentified by-products.

g Reaction conditions: (1) TMSOTf (2 equiv.), CH_2_Cl_2_, 0 °C, 1 h. (2) CDI (1.5 equiv.), CH_2_Cl_2_, 0 °C – rt, overnight.^7^

It is noteworthy that the reaction can be performed on a gram scale without affecting the yield and both isomers are easily separable by FCC (see the ESI†).

The ^1^H and ^13^C NMR data of the major *E* isomer 16a were consistent with those published previously.^8^ Possible racemization in the course of the reaction was dismissed based on the comparing specific optical rotation with the published data for 16a ([*α*]^22^_D_ = −261.1 (*c* 1.01, MeOH); ref. 8: [*α*]^22^_D_ = −207 (*c* 1.0, MeOH)). Most importantly, X-ray crystallographic analysis of 16a (Fig. 2; see the ESI† for further details)^14^ confirmed its absolute configuration on the C-7a carbon atom.

**Fig. 2:**
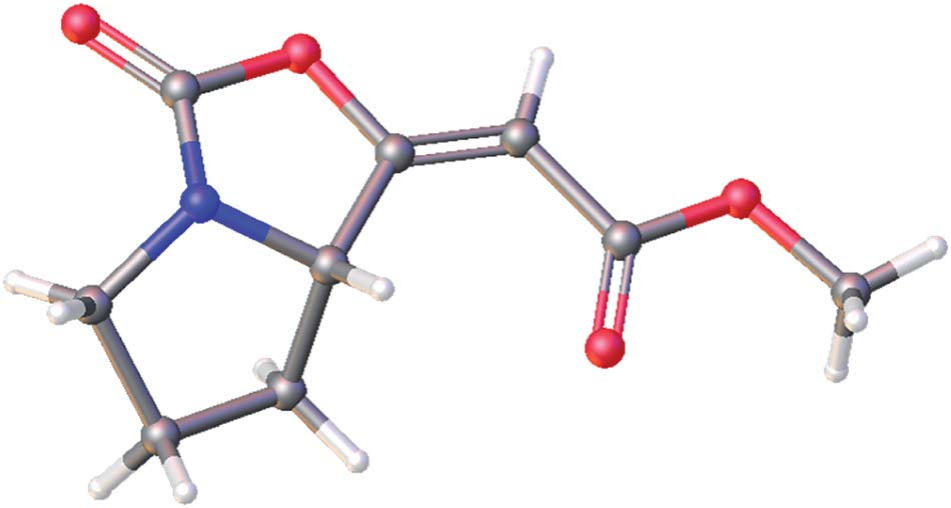
Molecular structure of the enol-carbamate *E*-16a confirmed by X-ray crystallographic analysis.

The minor *Z* isomer 16b was isolated for the first time as the pure compound and was fully characterized. Its structure was assigned on the basis of its ^1^H, ^13^C, COSY, HSQC, and HMBC NMR spectra.

A plausible mechanism of the cyclization of β-ketoester 15 was based upon previous works^12*a*–*c*^ and it is depicted in Scheme 2. Isocyanate cation II, as a key intermediate, can be formed directly from iminium triflate I (path A) or through the formation of carbamoyl triflate III with subsequent elimination of triflate ion spontaneously (path B). Ester enolate moiety IV then reacts as *O*-nucleophile *via* 5-*endo-dig* cyclization and leads predominantly to the formation of the enol-carbamate 16a.

**Scheme 2:**
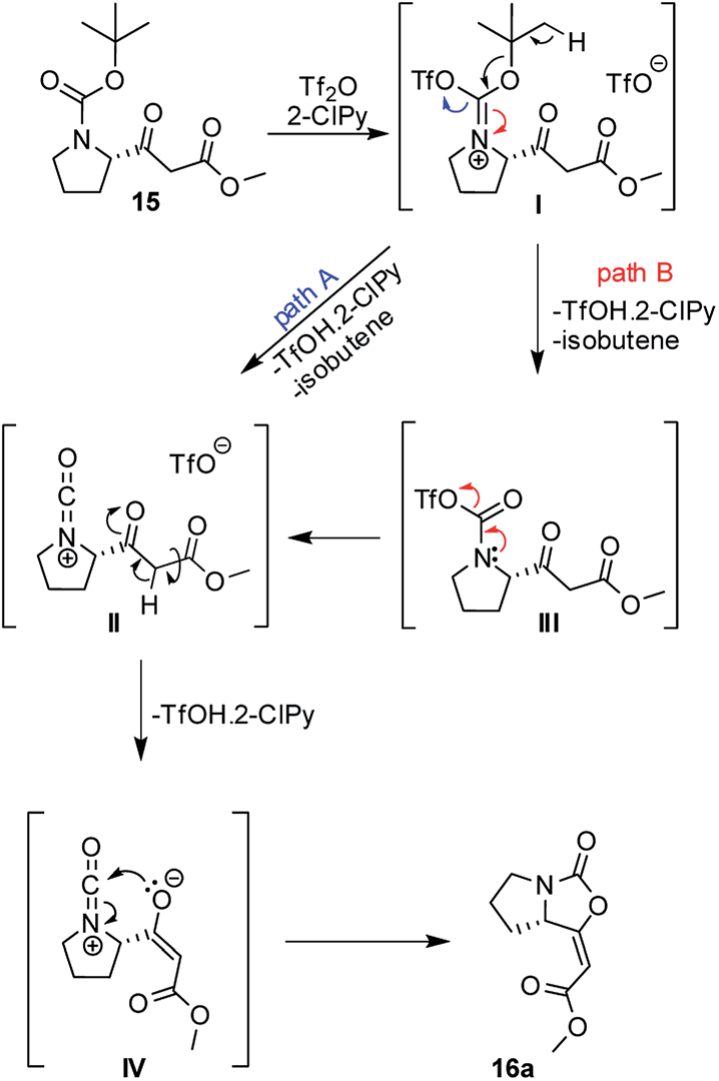
Plausible mechanism of the cyclization β-ketoester 15.

Next, the optimized conditions were briefly applied in the synthesis of the brabantamide A analogue 21 (Scheme 3). Starting β-ketoester 18 was synthetized in two steps in a 70% yield using both commercially available *N*-Boc-d-proline 17 and 2-(trimethylsilyl)ethanol. It ought to be mentioned that previously examined hydrolysis of the corresponding methyl ester 16a under acidic as well as basic conditions failed due to the instability of the bicyclic enol-carbamate.^3,8^

**Scheme 3:**
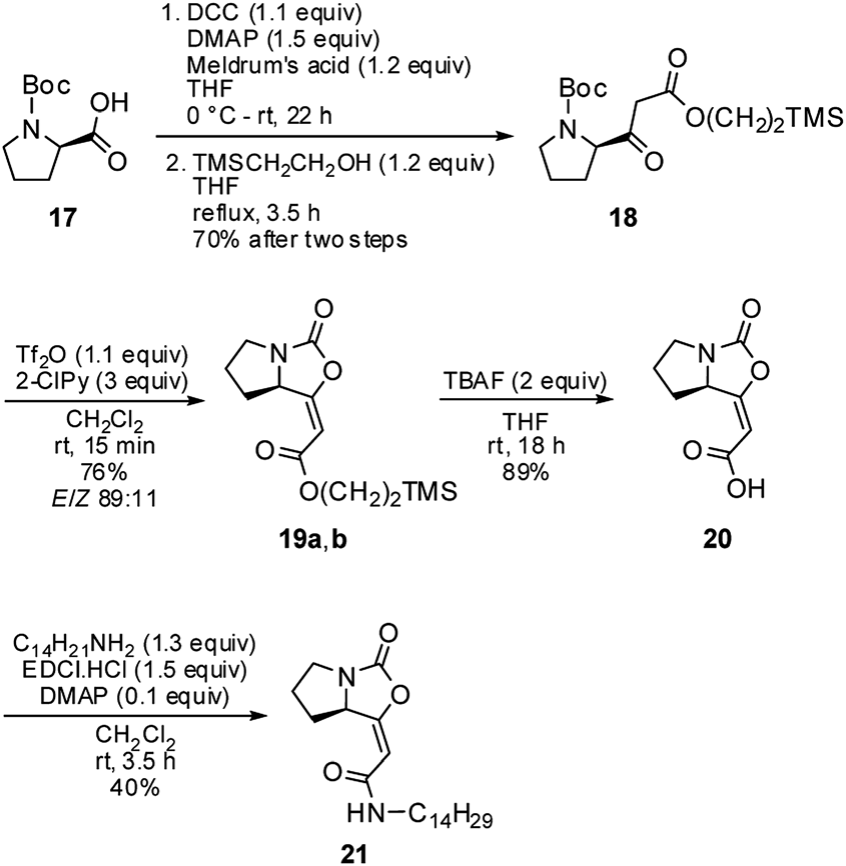
Synthesis of the brabantamide A analogue 21.

Subsequent cyclization of ester 18 using optimized reaction conditions afforded enol-carbamate 19 in 76% yield as a mixture of *E* and *Z* isomers in a ratio of 89 : 11. After isolation of the major isomer *E*-19a, it was treated with TBAF, providing free acid 20 in 89% yield. Finally, an amidation of 20 with tetradecylamine in the presence of EDCI gave amide 21 in moderate 40% yield. Both free acid 20 and amide 21 were fully characterized for the first time and their structures were assigned on the basis of its ^1^H, ^13^C, COSY, HSQC, and HMBC NMR spectra. Moreover, their structures were unambiguously confirmed by X-ray crystallographic analysis (Fig. 3; see ESI† for further details).^14^

**Fig. 3:**
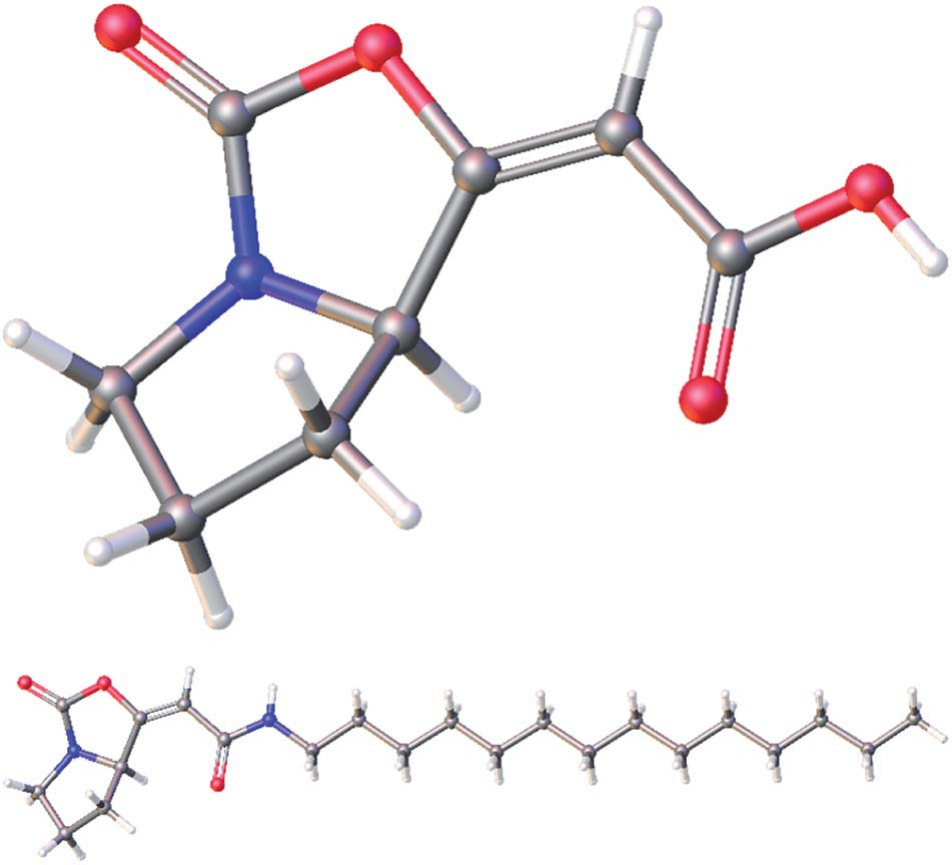
Molecular structures of acid 20 (top) and amide 21 (bottom) confirmed by X-ray crystallographic analysis.

## Conclusions

In conclusion, a new method of preparing bicyclic enol-carbamates with exocyclic double bond has been developed. Bicyclic oxazolidinone framework was obtained in one step from readily available β-ketoesters in very good yields and with high *E*/*Z* selectivity under mild reaction conditions using Tf_2_O and 2-chloropyridine tandem. The simplicity of this method was exemplified by a short and effective synthesis of the analogue of brabantamide A from commercially available *N*-Boc-d-proline in five steps with an overall 17% yield.

## Conflicts of interest

There are no conflicts to declare.

## Supplementary Material

RA-010-D0RA00796J-s001

RA-010-D0RA00796J-s002
