# Supplementary material for: Simple and efficient synthesis of bicyclic enol-carbamates: access to brabantamides and their analogues
Source: RSC Adv. 2020 Feb 13;10(12):6790–3. doi: 10.1039/d0ra00796j (PMC9049743; doi:10.1039/d0ra00796j)
Supplement: RA-010-D0RA00796J-s001 [file RA-010-D0RA00796J-s001.pdf]

Electronic Supplementary Material (ESI) for RSC Advances.
This journal is © The Royal Society of Chemistry 2020

Supporting Information

Simple and efficient synthesis of bicyclic enol-carbamates: Access to brabantamides and their analogues

Ondrej Záborský,*,† Ľudmila Petrovičová,† Jana Doháňošová,‡ Ján Moncoľ,§ and Róbert Fischer†

†Institute of Organic Chemistry, Catalysis and Petrochemistry, Slovak University of Technology in Bratislava, Radlinského 9, 81237, Bratislava, Slovak Republic

‡Central Laboratories, Slovak University of Technology in Bratislava, Radlinského 9, 81237, Bratislava, Slovak Republic

§Institute of Inorganic Chemistry, Technology and Materials, Slovak University of Technology in Bratislava, Radlinského 9, 81237, Bratislava, Slovak Republic

Table of Contents

1. General Information S2
2. General Procedures and Characterization of the Synthesized Compounds S2–S7
3. ^{1}H, ^{13}C, COSY and HSQC Spectra of the Synthesized Compounds S8–S23
4. X-Ray Structure Analyses of Compounds **16a**, **20** and **21** S24–S25
5. References and Notes S26

General Information: Flash column chromatography (FCC) was carried out with a Büchi system (Pump Manager C-615 and Fraction Collector C-660) using Normasil 60 silica gel (0.040–0.063 mm; VWR). Thin Layer Chromatography (TLC) analysis was carried out using TLC Silica gel 60 F_{254} (aluminium sheets, Merck), and plates were visualized with UV light or by treatment with permanganate solution followed by heating. Optical rotations were measured with a JASCO P-2000 digital polarimeter with a Na-D lamp (10 cm cell length). Concentrations (*c*) are given in grams per 100 mL. Melting points were measured with a Melting Point B-540 apparatus (Büchi). Infrared (IR) spectra were recorded as neat samples with a Nicolet 5700 FTIR spectrometer with an ATR Smart Orbit Diamond adapter (Thermo Electron Corporation). NMR spectra were recorded with a Varian VNMRS-600 instrument (^{1}H, 599.75 MHz, and ^{13}C, 150.81 MHz) in $CDCl_3$ (using tetramethylsilane as the internal standard). Data are presented as follows: chemical shift, multiplicity (s = singlet, bs = broad singlet, d = doublet, t = triplet, q = quartet, dd = doublet of doublets, dq = doublet of quartets, ddd = doublet of doublet of doublets, dddd = doublet of doublet of doublet of doublets, dtd = doublet of triplet of doublets, td = triplet of doublets, dt = doublet of triplets, m = multiplet), coupling constants (*J*/Hz) and integration. ^{1}H and ^{13}C signals were assigned by 2D experiments (COSY, HSQC and HMBC). HRMS analyses were carried out with an Orbitrap Velos Pro spectrometer (Thermo Fisher Scientific). All solvents used were dried and distilled according to conventional methods. *N*-Boc-L-proline and *N*-Boc-D-proline were commercially available.

Preparation of *β*-Ketoester 15:[1]

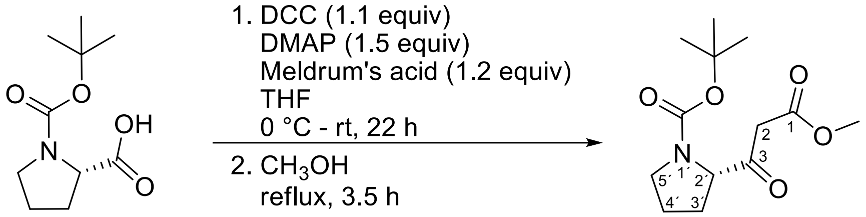

22 **15**

N-Boc-L-proline (**22**) (1.5 g, 7.0 mmol, 1 equiv) was dissolved in anhydrous THF (18 mL), the reaction mixture was cooled to 0 °C (ice bath) and DCC (1.57 g, 7.7 mmol, 1.1 equiv) was added. After stirring for 2 min, DMAP (1.28 g, 10.5 mmol, 1.5 equiv) and Meldrum's acid (1.21 g, 8.4 mmol, 1.2 equiv) were added. The reaction mixture was stirred for 22 h with a gradual warming to a room temperature. After this time, the suspension was filtered and the filtrate was concentrated under reduced pressure. The residue was dissolved in CH_2Cl_2 (50 mL), washed with 1 M HCl (2×10 mL) and finally with water (1×20 mL). The organic layer was dried over anhydrous $MgSO_4$, filtered and the solvent was evaporated under reduced pressure. The Meldrum's acid intermediate as a yellow foam was dissolved in MeOH (30 mL) and the reaction mixture was stirred under reflux for 3.5 hours. Then the reaction mixture was concentrated under reduced pressure and the product was isolated by FCC (hexanes/EtOAc, 85:15) to give *β*-ketoester **15** as a colourless oil in 79% yield (1.5 g, 5.5 mmol).

(–)-Methyl (2'*S*)-3-(*N*-*tert*-butoxycarbonyl-2'-pyrrolidinyl)-3-oxopropanoate (15):

$\mathbf{R_f}$ (*n*-hexane:EtOAc, 1:1) = 0.36. $[\alpha]_D^{25}$ = –69.4 (*c* 0.99, acetone).

^{1}H NMR (600 MHz, $CDCl_3$) mixture of rotamers (57:43), δ/ppm (major): 4.28 (dd, *J*= 8.5, 5.8 Hz, 1H, H-2'), 3.75 (s, 3H, OCH_3), 3.60–3.54 (m, 1H, H-5'), 3.52 (d, *J*= 16.0 Hz, 1H, H-2), 3.50 (d, *J*= 16.1 Hz, 1H, H-2), 3.50–3.45 (m, 1H, H-5'), 2.25–2.19 (m, 1H, H-3'), 2.00–1.95 (m, 1H, H-3'), 1.95–1.83 (m, 2H, H-4'), 1.43 (s, 9H, $C(CH_3)_3$); δ/ppm (minor): 4.39 (dd, *J*= 8.3, 4.9 Hz, 1H, H-2'), 3.73 (s, 3H, OCH_3), 3.62 (d, *J*= 15.8 Hz, 1H, H-2), 3.57 (d, *J*= 15.7 Hz, 1H, H-2), 3.45–3.40 (m, 2H, H-5'), 2.15–2.08 (m, 1H, H-3'), 2.07–2.01 (m, 1H, H-3'), 1.95–1.83 (m, 2H, H-4'), 1.47 (s, 9H, $C(CH_3)_3$).

^{13}C NMR (150 MHz, $CDCl_3$) mixture of rotamers, δ/ppm (major): 202.7 (C-3), 167.5 (C-1), 153.9 (CO), 80.9 ($\underline{C}(CH_3)_3$), 65.7 (C-2'), 52.5 (OCH_3), 46.9 (C-5'), 45.1 (C-2), 29.9 (C-3'), 28.3 ($C(\underline{C}H_3)_3$),

23.9 (C-4’); δ/ppm (minor): 203.1 (C-3), 167.8 (C-1), 154.9 (CO), 80.3 (<u>C</u>(CH3)3), 65.2 (C-2’), 52.4 (OCH_3), 47.1 (C-5’), 46.2 (C-2), 28.7 (C-3’), 28.5 (C(<u>C</u>H3)3), 24.6 (C-4’).

Cyclization of *β*-Ketoester 15:

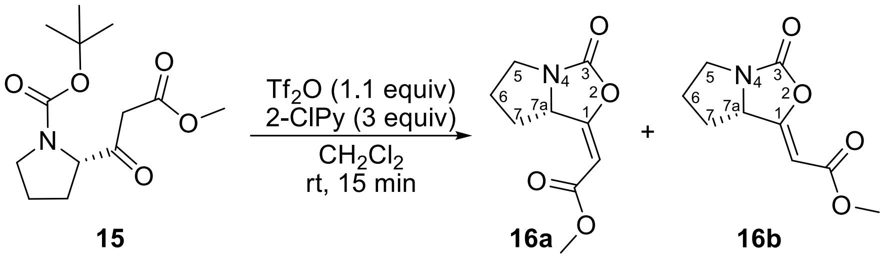

To a stirred solution of *β*-ketoester **15** (1.043 g, 3.84 mmol, 1 equiv) in anhydrous CH_2Cl_2 (38 mL) was added 2-chloropyridine (1.09 mL, 11.52 mmol, 3 equiv) at room temperature under Ar atmosphere, followed by trifluoromethanesulfonic anhydride (0.71 mL, 4.22 mmol, 1.1 equiv). The reaction progress was monitored by TLC (CH_2Cl_2:EtOAc, 7:3) and after the consumption of the starting material (15 minutes), the reaction mixture was filtered through the pad of silica gel (CH_2Cl_2:EtOAc, 9:1 as the eluent). The filtrate was concentrated under reduced pressure and the excess of 2-chloropyridine was removed by using Kugelrohr distillation (50 °C, <1 Torr). The residue was purified by FCC (CH_2Cl_2:EtOAc, 95:5) to give two pure isomers *E*-**16a** and *Z*-**16b** in total yield of 81% (615 mg, 3.12 mmol) with the ratio of **16a**:**16b** 85:15.

(–)-(*S*, *E*)-Methyl 2-(3-oxotetrahydropyrrolo[1,2-*c*]oxazol-1(3*H*)-ylidene)acetate (16a):

Yield: 69% (524 mg, 2.66 mmol). Colourless solid. **R$_f$** (CH_2Cl_2:EtOAc, 7:3) = 0.60. **mp** = 48–50 °C. $[\alpha]_D^{22}$ = –261.1 (*c* 1.01, methanol).

^{1}H NMR (600 MHz, $CDCl_3$) δ/ppm: 5.67 (d, *J* = 2.1 Hz, 1H, C=CH), 4.92 (ddd, *J*= 9.4, 6.7, 2.0 Hz, 1H, H-7a), 3.73 (s, 3H, OCH_3), 3.69 (dt, *J* = 11.4, 7.9 Hz, 1H, H-5), 3.30 (ddd, *J* = 11.4, 8.9, 4.6 Hz, 1H, H-5), 2.63 (dddd, *J* = 12.7, 7.2, 6.8, 3.2 Hz, 1H, H-7), 2.17–2.04 (m, 2H, H-6), 1.60 (dddd, *J* = 12.7, 9.7, 9.4, 9.0 Hz, 1H, H-7).

^{13}C NMR (150 MHz, $CDCl_3$) δ/ppm: 166.7 (CO), 165.8 (C-1), 156.8 (C-3), 95.4 (C=<u>C</u>H), 64.3 (C-7a), 51.6 (OCH_3), 46.1 (C-5), 30.4 (C-7), 26.4 (C-6).

(–)-(*S*, *Z*)-Methyl 2-(3-oxotetrahydropyrrolo[1,2-*c*]oxazol-1(3*H*)-ylidene)acetate (16b):

Yield: 12% (91 mg, 0.46 mmol). Colourless solid. **R$_f$** (CH_2Cl_2:EtOAc, 7:3) = 0.45. **mp** = 60–62 °C. $[\alpha]_D^{25}$ = –73.2 (*c* 1.01, methanol). **HRMS (ESI)**: *m*/*z* calcd. for $C_9H_{12}NO_4$ $[M+H]^+$: 198.0760, found: 198.0761. **IR** (ATR): ν = 1789, 1716, 1676, 1378, 1197, 1173, 1142, 1008, 967, 822, 764 cm^{-1}.

^{1}H NMR (600 MHz, $CDCl_3$) δ/ppm: 5.15 (d, *J* = 1.6 Hz, 1H, C=CH), 4.48 (ddd, *J*= 9.2, 6.9, 1.5 Hz, 1H, H-7a), 3.75 (s, 3H, OCH_3), 3.68 (dt, *J* = 11.3, 7.8 Hz, 1H, H-5), 3.31 (ddd, *J* = 11.3, 8.9, 4.4 Hz, 1H, H-5), 2.25 (dddd, *J*= 12.4, 7.2, 6.9, 3.2 Hz, 1H, H-7), 2.20–2.13 (m, 1H, H-6), 2.12–2.03 (m, 1H, H-6), 1.72 (dddd, *J* = 12.4, 9.8, 9.2, 8.5 Hz, 1H, H-7).

^{13}C NMR (150 MHz, $CDCl_3$) δ/ppm: 164.2 (CO), 160.8 (C-1), 157.2 (C-3), 94.3 (C=<u>C</u>H), 63.7 (C-7a), 51.7 (OCH_3), 46.2 (C-5), 31.5 (C-7), 26.5 (C-6).

Preparation of *β*-Ketoester 18:

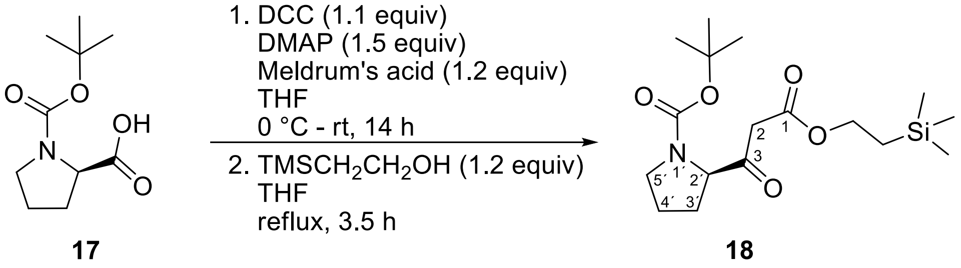

N-Boc-D-proline (**17**) (1.9 g, 8.8 mmol, 1 equiv) was dissolved in anhydrous THF (23 mL), the solution was cooled to 0 °C (ice bath) and DCC (2.0 g, 9.7 mmol, 1.1 equiv) was added. After stirring for 2 min, DMAP (1.6 g, 13.2 mmol, 1.5 equiv) and Meldrum's acid (1.5 g, 10.6 mmol, 1.2 equiv) were added. The reaction mixture was stirred for 14 h with a gradual warming to a room temperature. After this time, the suspension was filtered and the filtrate was concentrated under reduced pressure. The residue mixture was dissolved in CH_2Cl_2 (50 mL), washed with 1 M HCl (2×15 mL) and finally with water (1×30 mL). The organic layer was dried over anhydrous $MgSO_4$, filtered and the solvent was evaporated under reduced pressure. The Meldrum's acid intermediate as a yellow foam was dissolved in THF (24 mL), 2-(trimethylsilyl)ethanol (1.52 mL, 10.6 mmol, 1.2 equiv) was added and the reaction mixture was stirred under reflux for 3.5 hours. Then the reaction mixture was concentrated under reduced pressure and the product was isolated by FCC (hexanes:EtOAc, 90:10) to give *β*-ketoester **18** as a colourless oil in 70% yield (2.2 g, 6.2 mmol).

(+)-2-(Trimethylsilyl)ethyl (2'*R*)-3-(*N*-*tert*-butoxycarbonyl-2'-pyrrolidinyl)-3-oxopropanoate (18):

R$_f$ (*n*-hexane:EtOAc, 1:1) = 0.52. $[\alpha]_D^{25}$ = +55.0 (*c* 1.10, acetone). **HRMS (ESI)**: *m*/*z* calcd. for $C_{17}H_{32}NO_5Si$ $[M+H]^+$: 358.2049, found: 358.2042. **IR** (ATR): ν = 1746, 1694, 1390, 1366, 1249, 1161, 1115, 856, 835, 770 cm^{-1}.

^{1}H NMR (600 MHz, $CDCl_3$) mixture of rotamers (59:41), δ/ppm (major): 4.28 (dd, *J* = 8.4, 5.8 Hz, 1H, H-2'), 4.25–4.20 (m, 2H, OCH_2), 3.60–3.53 (m, 1H, H-5'), 3.52–3.45 (m, 3H, H-2, H-5'), 2.25–2.18 (m, 1H, H-3'), 2.01–1.95 (m, 1H, H-3'), 1.95–1.82 (m, 2H, H-4'), 1.44 (s, 9H, $C(CH_3)_3$), 1.04–0.99 (m, 2H, $SiCH_2$), 0.04 (s, 9H, $Si(CH_3)_3$); δ/ppm (minor): 4.39 (dd, *J* = 8.3, 4.9 Hz, 1H, H-2'), 4.24–4.19 (m, 2H, OCH_2), 3.60–3.53 (m, 2H, H-2), 3.43 (t, *J*= 6.9 Hz, 2H, H-5'), 2.14–2.07 (m, 1H, H-3'), 2.07–2.01 (m, 1H, H-3'), 1.95–1.82 (m, 2H, H-4'), 1.46 (s, 9H, $C(CH_3)_3$), 1.04–0.99 (m, 2H, $SiCH_2$), 0.04 (s, 9H, $Si(CH_3)_3$).

^{13}C NMR (150 MHz, $CDCl_3$) mixture of rotamers, δ/ppm (major): 202.9 (C-3), 167.3 (C-1), 153.9 (CO), 80.8 ($\underline{C}(CH_3)_3$), 65.7 (C-2'), 63.9 (OCH_2), 46.9 (C-5'), 45.6 (C-2), 29.9 (C-3'), 28.4 ($C(\underline{C}H_3)_3$), 23.9 (C-4'), 17.4 ($SiCH_2$), -1.4 ($Si(CH_3)_3$); δ/ppm (minor): 203.2 (C-3), 167.6 (C-1), 154.9 (CO), 80.3 ($\underline{C}(CH_3)_3$), 65.2 (C-2'), 63.7 (OCH_2), 47.1 (C-5'), 46.7 (C-2), 28.7 (C-3'), 28.5 ($C(\underline{C}H_3)_3$), 24.6 (C-4'), 17.4 ($SiCH_2$), -1.4 ($Si(CH_3)_3$).

Cyclization of *β*-Ketoester 18:

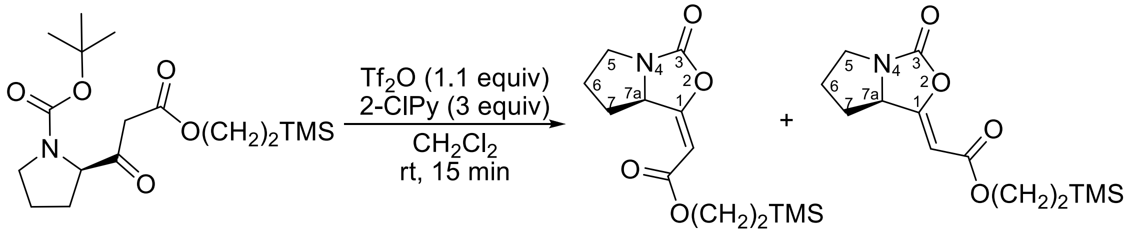

To a stirred solution of *β*-ketoester **18** (1.213 g, 3.4 mmol, 1 equiv) in anhydrous CH_2Cl_2 (34 mL) was added 2-chloropyridine (1 mL, 10.2 mmol, 3 equiv) at room temperature under Ar atmosphere, followed by trifluoromethanesulfonic anhydride (0.63 mL, 3.74 mmol, 1.1 equiv). The reaction progress was monitored by TLC (CH_2Cl_2:EtOAc, 7:3) and after the consumption of the starting material (15 minutes), the reaction mixture was filtered through the pad of silica gel (CH_2Cl_2:EtOAc, 9:1 as the eluent). The filtrate was concentrated under reduced pressure and the excess of 2-chloropyridine was removed by using Kugelrohr distillation (50 °C, <1 Torr). The residue was purified by FCC (CH_2Cl_2 to CH_2Cl_2:EtOAc, 95:5)[2] to give two pure isomers *E*-**19a** and *Z*-**19b** in total yield of 76% (729 mg, 2.57 mmol) with the ratio of **19a**:**19b** 89:11.

(+)-(*R*, *E*)-2-(Trimethylsilyl)ethyl 2-(3-oxotetrahydropyrrolo[1,2-*c*]oxazol-1(3*H*)-ylidene)acetate (19a):

Yield: 67% (650 mg, 2.29 mmol). Colourless solid. **R_f** (CH_2Cl_2:EtOAc, 7:3) = 0.67. **mp** = 36–38 °C. $[\alpha]_D^{22}$ = +191.9 (*c* 0.90, methanol).

^{1}H NMR (600 MHz, $CDCl_3$) δ/ppm: 5.64 (d, *J* = 2.0 Hz, 1H, C=CH), 4.91 (ddd, *J*= 9.4, 6.6, 2.0 Hz, 1H, H-7a), 4.26–4.18 (m, 2H, OCH_2), 3.69 (dt, *J* = 11.4, 7.9 Hz, 1H, H-5), 3.30 (ddd, *J* = 11.4, 8.9, 4.6 Hz, 1H, H-5), 2.63 (dddd, *J* = 12.7, 7.2, 6.7, 3.2 Hz, 1H, H-7), 2.16–2.03 (m, 2H, H-6), 1.60 (dddd, *J* = 12.7, 9.7, 9.5, 9.1 Hz, 1H, H-7), 1.05–0.99 (m, 2H, $SiCH_2$), 0.05 (s, 9H, $Si(CH_3)_3$).

^{13}C NMR (150 MHz, $CDCl_3$) δ/ppm: 166.5 (CO), 165.5 (C-1), 156.9 (C-3), 96.0 (C=<u>C</u>H), 64.3 (C-7a), 62.7 (OCH_2), 46.1 (C-5), 30.5 (C-7), 26.4 (C-6), 17.5 ($SiCH_2$), -1.4 ($Si(CH_3)_3$).

(+)-(*R*, *Z*)-2-(Trimethylsilyl)ethyl 2-(3-oxotetrahydropyrrolo[1,2-*c*]oxazol-1(3*H*)-ylidene)acetate (19b):

Yield: 9% (79 mg, 0.28 mmol). Colourless solid. **R_f** (CH_2Cl_2:EtOAc, 7:3) = 0.53. **mp** = 69–71 °C. $[\alpha]_D^{25}$ = +53.3 (*c* 1.01, methanol). **HRMS (ESI)**: *m*/*z* calcd. for $C_{13}H_{22}NO_4Si$ $[M+H]^+$: 284.1318, found: 284.1313. **IR** (ATR): ν = 1789, 1711, 1680, 1388, 1249, 1176, 1141, 1000, 828, 763 cm^{-1}.

^{1}H NMR (600 MHz, $CDCl_3$) δ/ppm: 5.12 (d, *J*= 1.6 Hz, 1H, C=CH), 4.46 (ddd, *J*= 9.2, 6.9, 1.5 Hz, 1H, H-7a), 4.27–4.23 (m, 2H, OCH_2), 3.68 (dt, *J* = 11.3, 7.8 Hz, 1H, H-5), 3.30 (ddd, *J* = 11.3, 8.9, 4.4 Hz, 1H, H-5), 2.23 (dddd, *J* = 12.4, 7.1, 6.9, 3.2 Hz, 1H, H-7), 2.19–2.12 (m, 1H, H-6), 2.10–2.01 (m, 1H, H-6), 1.71 (dddd, *J* = 12.4, 9.7, 9.2, 8.6 Hz, 1H, H-7), 1.07–1.02 (m, 2H, $SiCH_2$), 0.05 (s, 9H, $Si(CH_3)_3$).

^{13}C NMR (150 MHz, $CDCl_3$) δ/ppm: 164.0 (CO), 160.4 (C-1), 157.3 (C-3), 94.9 (C=<u>C</u>H), 63.7 (C-7a), 62.8 (OCH_2), 46.2 (C-5), 31.6 (C-7), 26.5 (C-6), 17.5 ($SiCH_2$), -1.4 ($Si(CH_3)_3$).

Deesterification of Bicyclic Enol-Carbamate 19a:

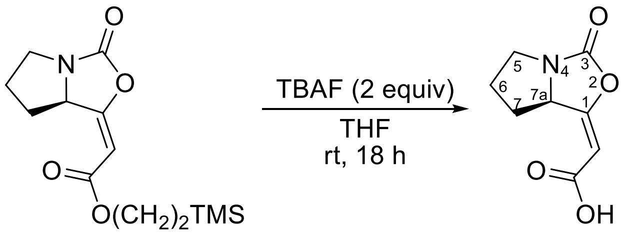

19a **20**

To a stirred solution of bicyclic enol-carbamate **19a** (650 mg, 2.30 mmol, 1 equiv) in anhydrous THF (9.2 mL) was added a solution of TBAF (1 M in THF, 4.6 mL, 4.6 mmol, 2 equiv) at room temperature under Ar atmosphere, and stirring was continued for 18 h. When TLC showed that the reaction was complete (CH_2Cl_2:EtOAc, 7:3), the reaction mixture was diluted with water (10 mL), acidified with 1 M HCl to pH = 3 and extracted with EtOAc (3×20 mL). The combined organic layers were dried over $MgSO_4$, filtered and concentrated under reduced pressure. The product was isolated by FCC (CH_2Cl_2 to CH_2Cl_2:MeOH:AcOH, 97:2.7:0.3)[3] to give acid **20** as a colourless solid in 89% yield (373 mg, 2.04 mmol).

(+)-(*R*, *E*)-2-(3-Oxotetrahydropyrrolo[1,2-*c*]oxazol-1(3*H*)-ylidene)acetic acid (20):

R$_f$ (CH_2Cl_2:MeOH:AcOH, 97:2.7:0.3) = 0.15. **mp** = decomp. >150 °C. **$[\alpha]_D^{25}$** = +167.6 (*c* 1.00, methanol). **HRMS (ESI)**: *m/z* calcd. for $C_8H_{10}NO_4$ $[M+H]^+$: 184.0610, found: 184.0607. **IR** (ATR): ν = 2905, 1794, 1637, 1154, 1028, 961, 885, 857, 750, 743 cm^{-1}.

^{1}H NMR (600 MHz, $CDCl_3$) δ/ppm: 10.61 (bs, 1H, COOH), 5.67 (d, *J* = 1.9 Hz, 1H, C=CH), 4.93 (ddd, *J* = 9.5, 6.7, 1.9 Hz, 1H, H-7a), 3.70 (dt, *J* = 11.4, 7.9 Hz, 1H, H-5), 3.31 (ddd, *J* = 11.4, 8.9, 4.5 Hz, 1H, H-5), 2.61 (dddd, *J* = 12.7, 7.0, 6.8, 3.1 Hz, 1H, H-7), 2.19–2.05 (m, 2H, H-6), 1.63 (dddd, *J* = 12.7, 9.9, 9.5, 9.5 Hz, 1H, H-7).

^{13}C NMR (150 MHz, $CDCl_3$) δ/ppm: 171.9, 168.0 (COOH, C-1), 156.5 (C-3), 95.3 (C=<u>C</u>H), 64.5 (C-7a), 46.1 (C-5), 30.3 (C-7), 26.4 (C-6).

Synthesis of Amide 21:

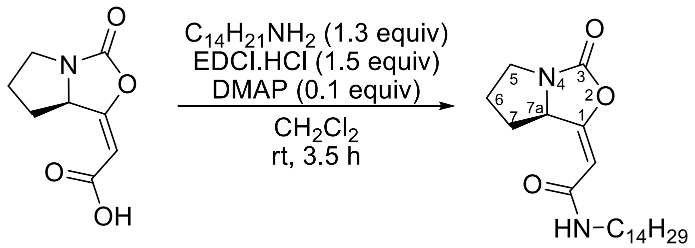

20 **21**

To a stirred solution of acid **20** (100 mg, 0.55 mmol, 1 equiv) in anhydrous CH_2Cl_2 (1.8 mL) were added EDCI.HCl (158 mg, 0.825 mmol, 1.5 equiv) and DMAP (6.7 mg, 0.055 mmol, 0.1 equiv) at room temperature under Ar atmosphere, and stirring was continued for 15 min. After this time, tetradecylamine (153 mg, 0.715 mmol, 1.3 equiv) was added and the reaction mixture was stirred for additional 3.5 h. When TLC showed that the reaction was complete (CH_2Cl_2:EtOAc, 7:3), it was quenched by the addition of 1 M HCl (3 mL) and the reaction mixture was extracted with EtOAc (3×10 mL). The combined organic layers were dried over $MgSO_4$, filtered and concentrated under reduced pressure. The residue was purified by FCC (hexanes:EtOAc, 75:25) to give amide **21** as a colourless solid in 40% yield (82 mg, 0.22 mmol).

(+)-(*R*, *E*)-2-(3-Oxotetrahydropyrrolo[1,2-*c*]oxazol-1(3*H*)-ylidene)-*N*-tetradecylacetamide (21):

R$_f$ (*n*-hexane:EtOAc, 1:1) = 0.34. **mp** = 66–68 °C. **$[\alpha]_D^{25}$** = +141.0 (*c* 1.01, chloroform). **HRMS (ESI)**: *m*/*z* calcd. for $C_{22}H_{39}N_2O_3$ $[M+H]^+$: 379.2961, found: 379.2960. **IR** (ATR): ν = 3292, 2918, 2850, 1797, 1682, 1614, 1541, 1470, 1160, 1035, 965 cm^{-1}.

^{1}H NMR (600 MHz, $CDCl_3$) δ/ppm: 5.69 (t, *J* = 5.4 Hz, 1H, NH), 5.57 (d, *J* = 2.0 Hz, 1H, C=CH), 4.99 (ddd, *J*= 9.5, 6.6, 2.0 Hz, 1H, H-7a), 3.66 (dt, *J* = 11.3, 7.9 Hz, 1H, H-5), 3.33–3.21 (m, 3H, H-5, $\underline{CH_2}$NH), 2.68 (dddd, *J* = 12.7, 7.0, 6.7, 3.1 Hz, 1H, H-7), 2.13–2.00 (m, 2H, H-6), 1.59 (dddd, *J* = 12.7, 9.7, 9.5, 9.1 Hz, 1H, H-7), 1.55–1.49 (m, 2H, $\underline{CH_2}CH_2NH$), 1.34–1.22 (m, 22H, $C_{11}\underline{H_{22}}CH_3$), 0.88 (t, *J* = 7.2 Hz, 3H, CH_3).

^{13}C NMR (150 MHz, $CDCl_3$) δ/ppm: 165.0, 162.2 (CO, C-1), 157.3 (C-3), 97.8 (C=$\underline{C}$H), 64.3 (C-7a), 46.0 (C-5), 39.6 (CH_2NH), 32.0 (CH_2), 31.0 (C-7), 5×29.8, 2×29.7, 29.5, 29.4, 27.1 (10×CH_2), 26.3 (C-6), 22.8 (CH_2), 14.2 (CH_3).

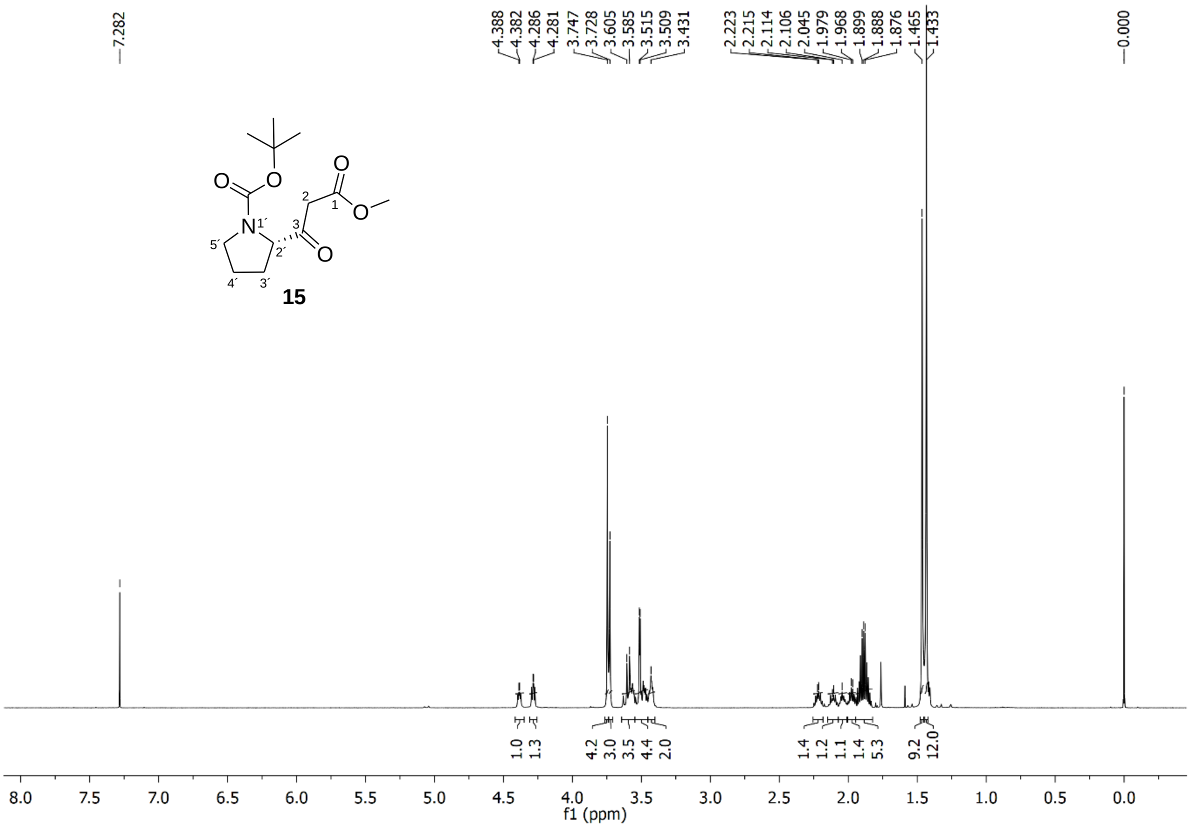
7.282
4.388
4.382
4.286
4.281
3.747
3.728
3.605
3.585
3.515
3.509
3.431
2.223
2.215
2.114
2.106
2.045
1.979
1.968
1.899
1.888
1.876
1.465
1.433
0.000
15
1.0
1.3
4.2
3.0
3.5
4.4
2.0
1.4
1.2
1.1
1.4
5.3
9.2
12.0
f1 (ppm)

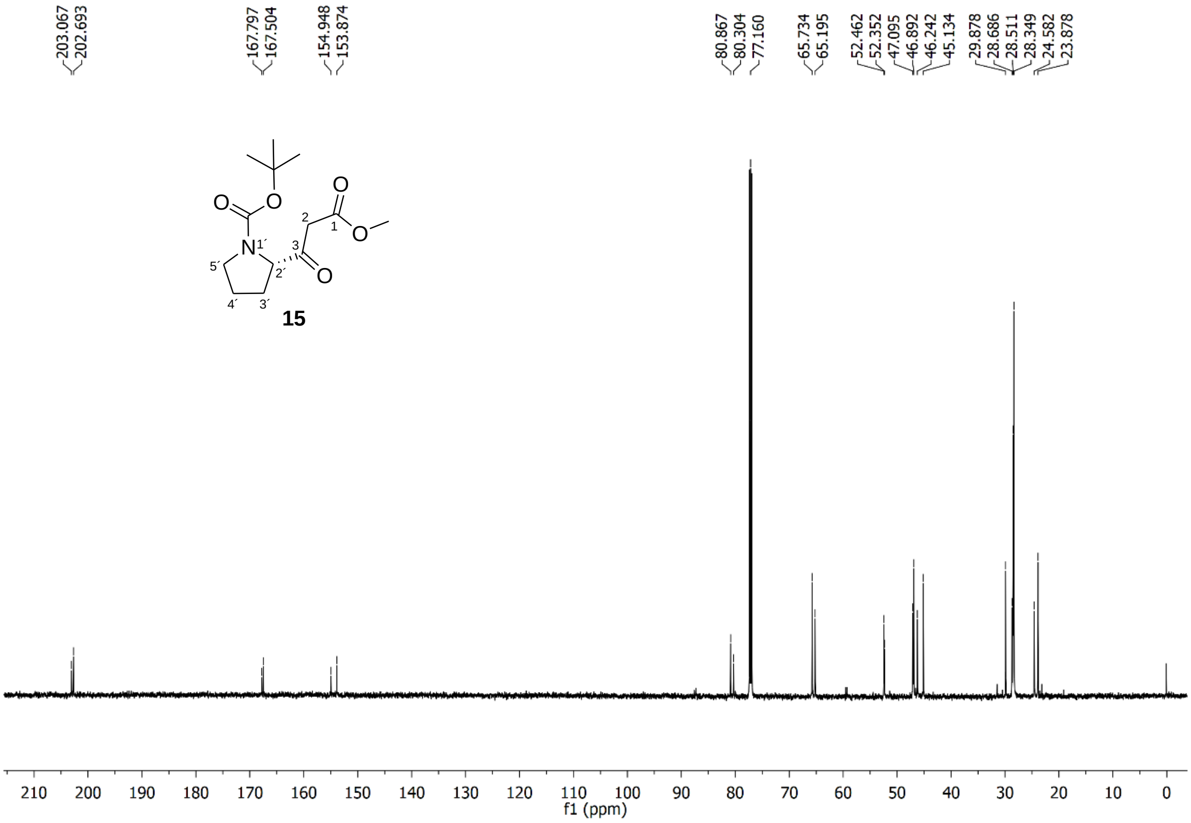
203.067
202.693
167.797
167.504
154.948
153.874
80.867
80.304
77.160
65.734
65.195
52.462
52.352
47.095
46.892
46.242
45.134
29.878
28.686
28.511
28.349
24.582
23.878
15
f1 (ppm)

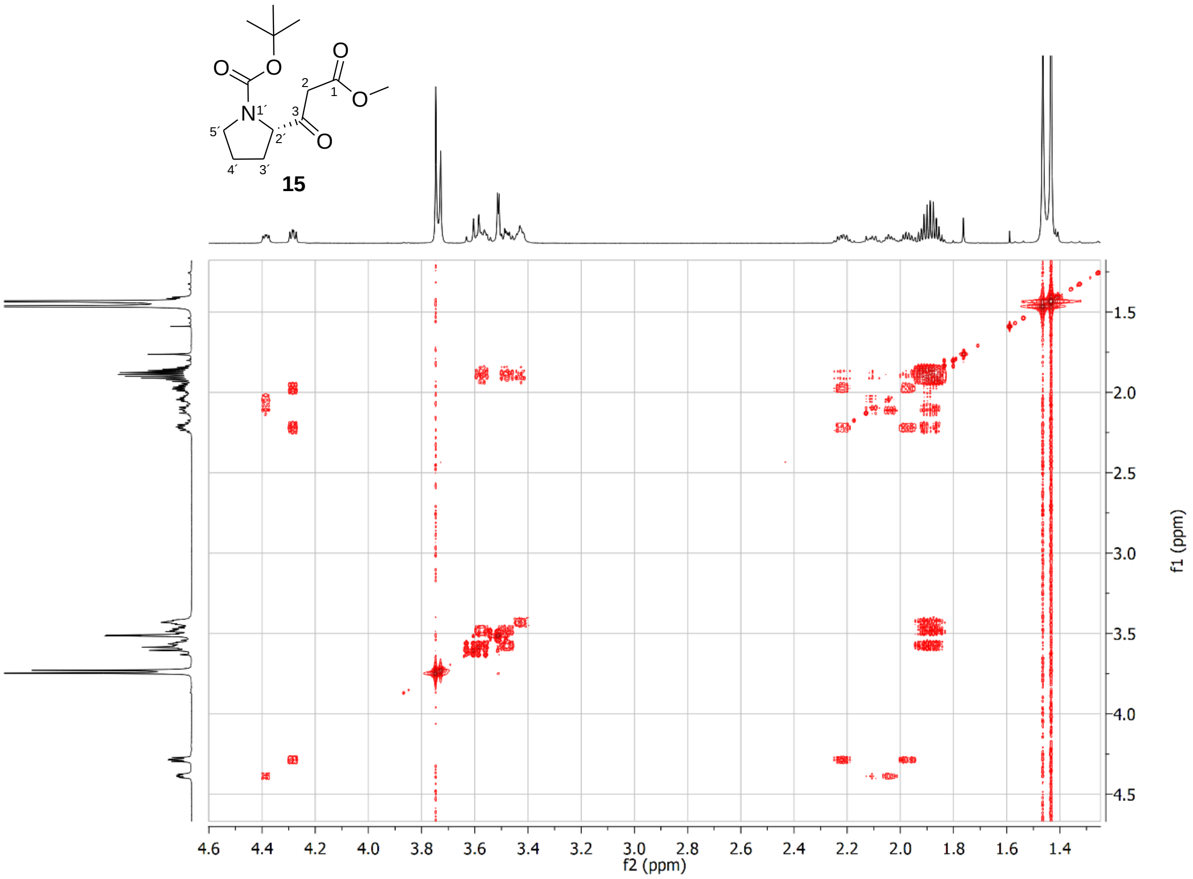
15
f1 (ppm)
f2 (ppm)

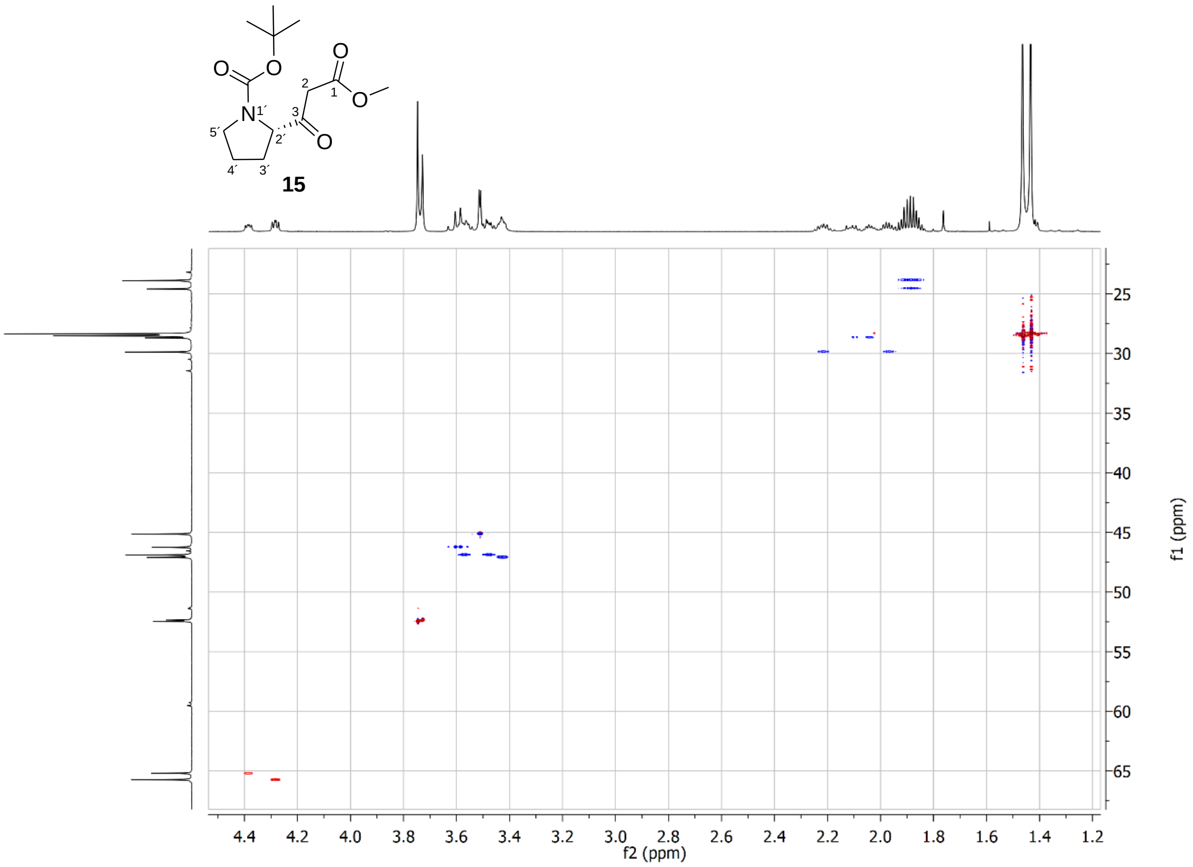
15
f1 (ppm)
f2 (ppm)

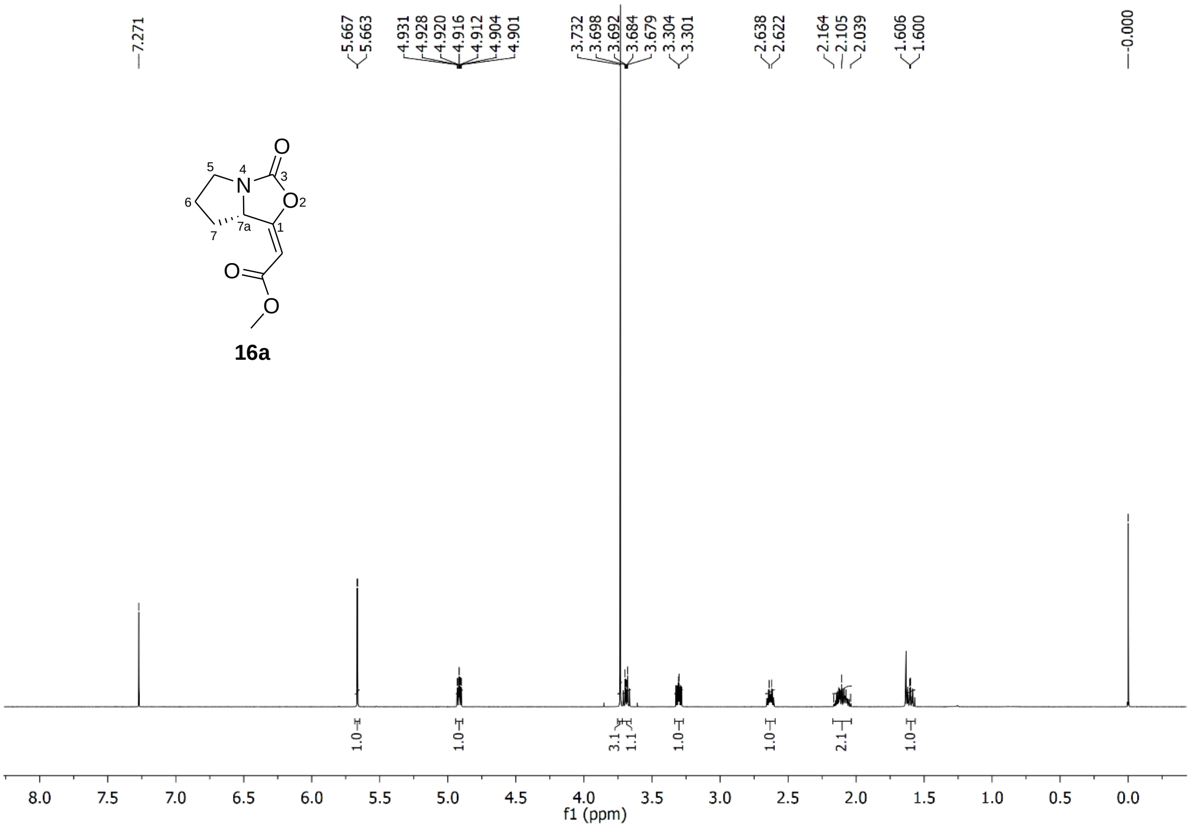
7.271
5.667
5.663
4.931
4.928
4.920
4.916
4.912
4.904
4.901
3.732
3.698
3.692
3.684
3.679
3.304
3.301
2.638
2.622
2.164
2.105
2.039
1.606
1.600
0.000
16a
1.0
1.0
3.1
1.1
1.0
1.0
2.1
1.0
f1 (ppm)

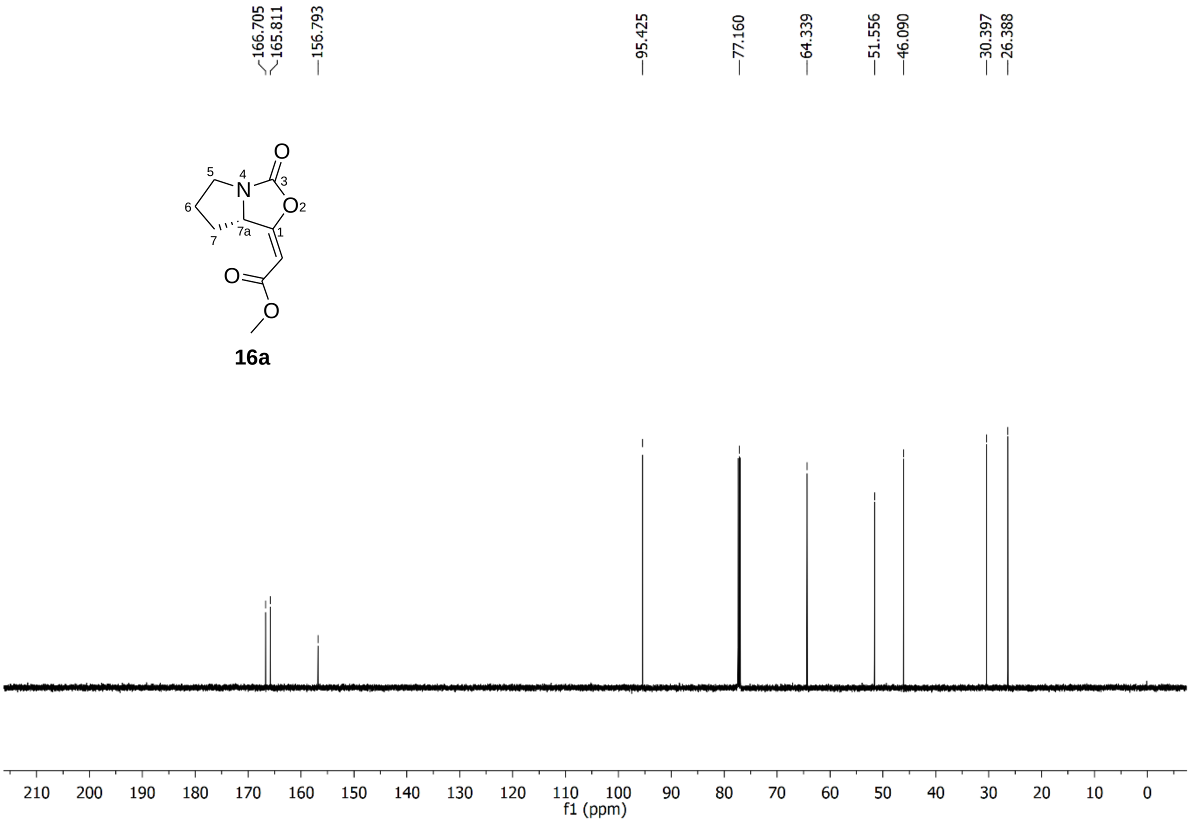
166.705
165.811
156.793
95.425
77.160
64.339
51.556
46.090
30.397
26.388
16a
f1 (ppm)

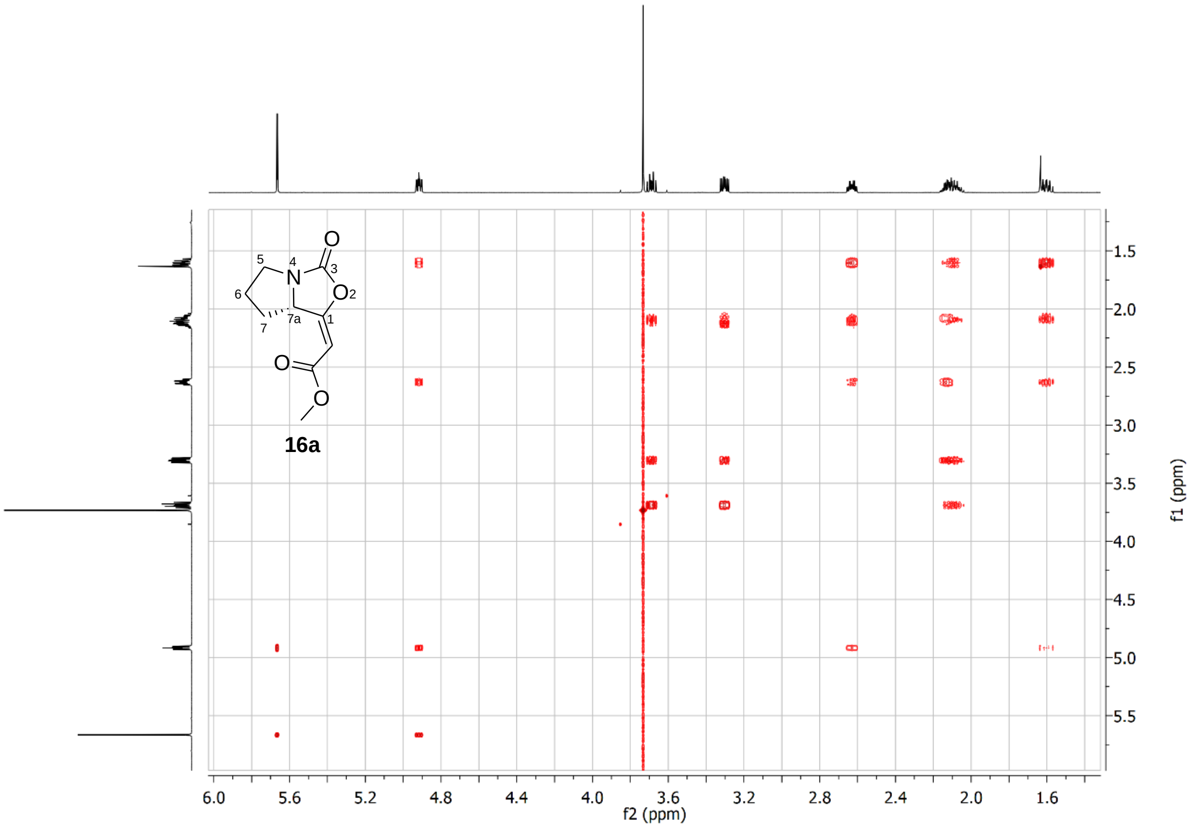
16a
f2 (ppm)
f1 (ppm)

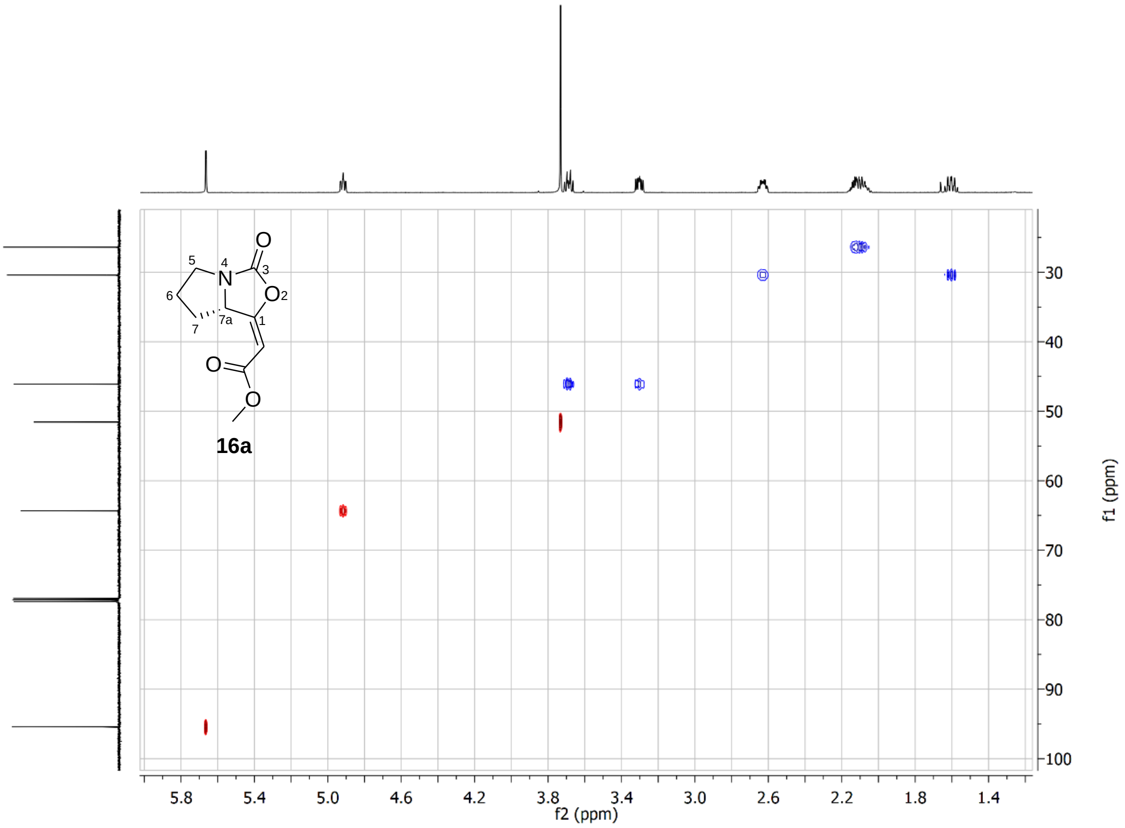
16a
f2 (ppm)
f1 (ppm)

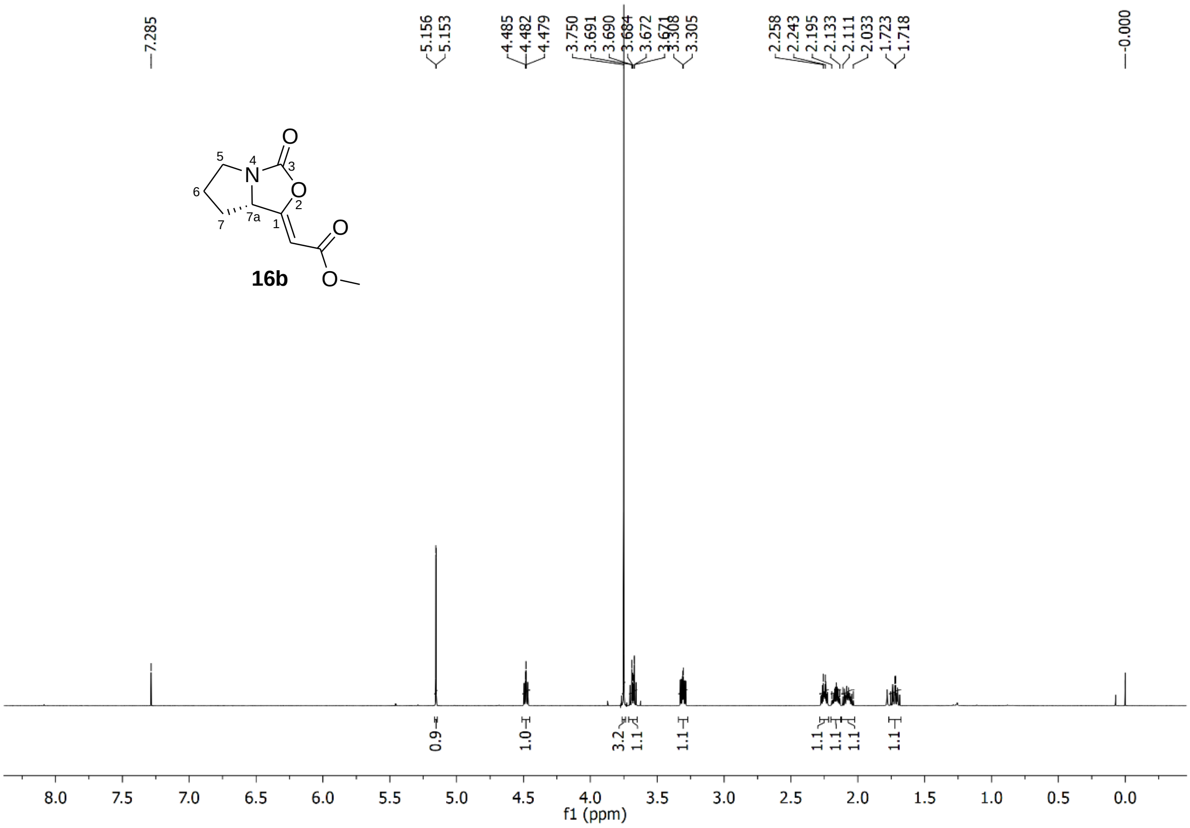
7.285
5.156
5.153
4.485
4.482
4.479
3.750
3.691
3.690
3.684
3.672
3.671
3.308
3.305
2.258
2.243
2.195
2.133
2.111
2.033
1.723
1.718
0.000
16b
0.9
1.0
3.2
1.1
1.1
1.1
1.1
1.1
1.1
f1 (ppm)

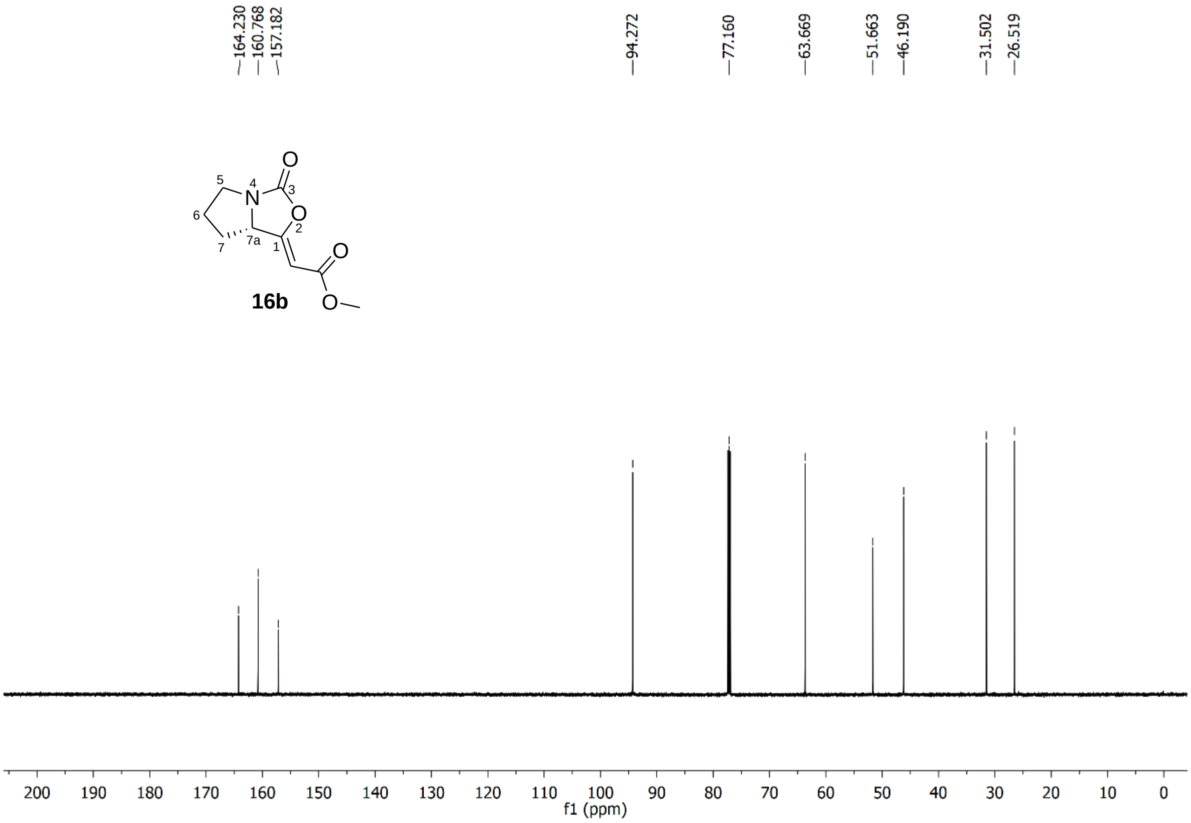
164.230
160.768
157.182
94.272
77.160
63.669
51.663
46.190
31.502
26.519
16b
f1 (ppm)

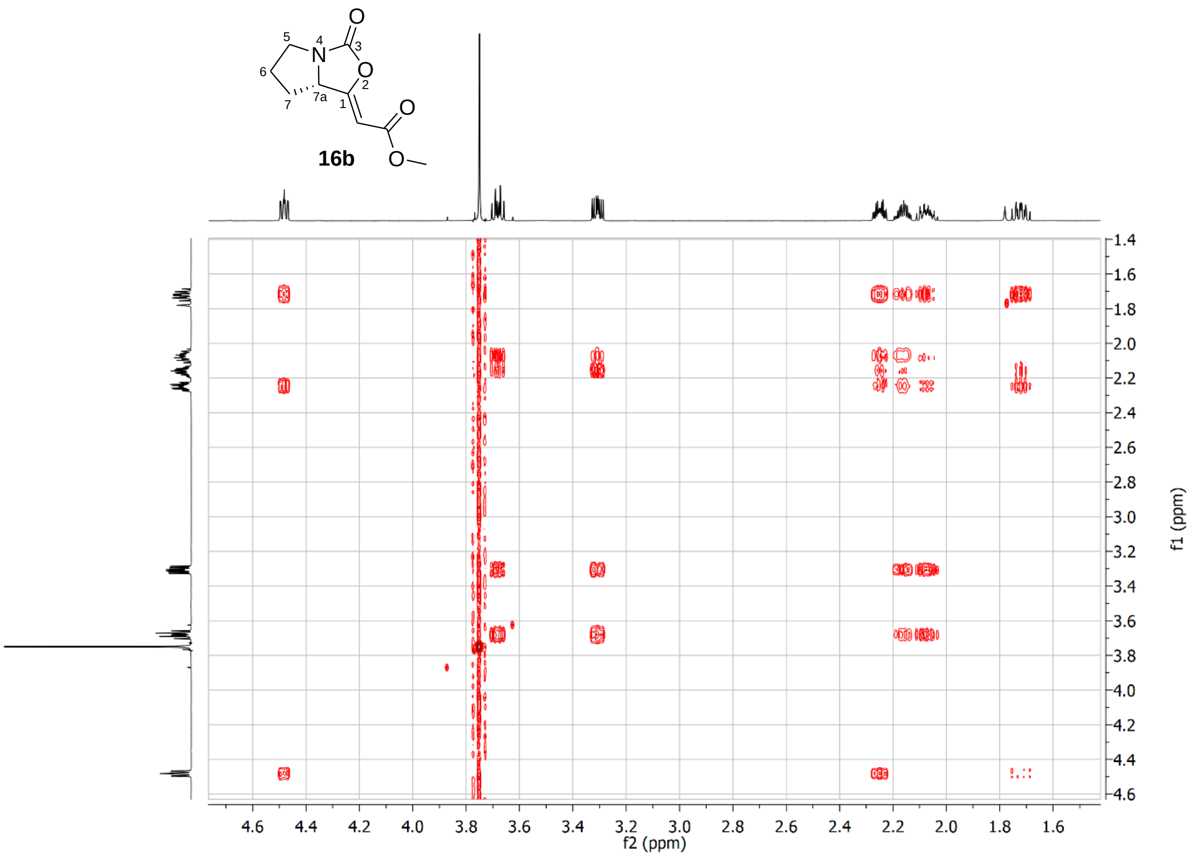
16b
f2 (ppm)
f1 (ppm)

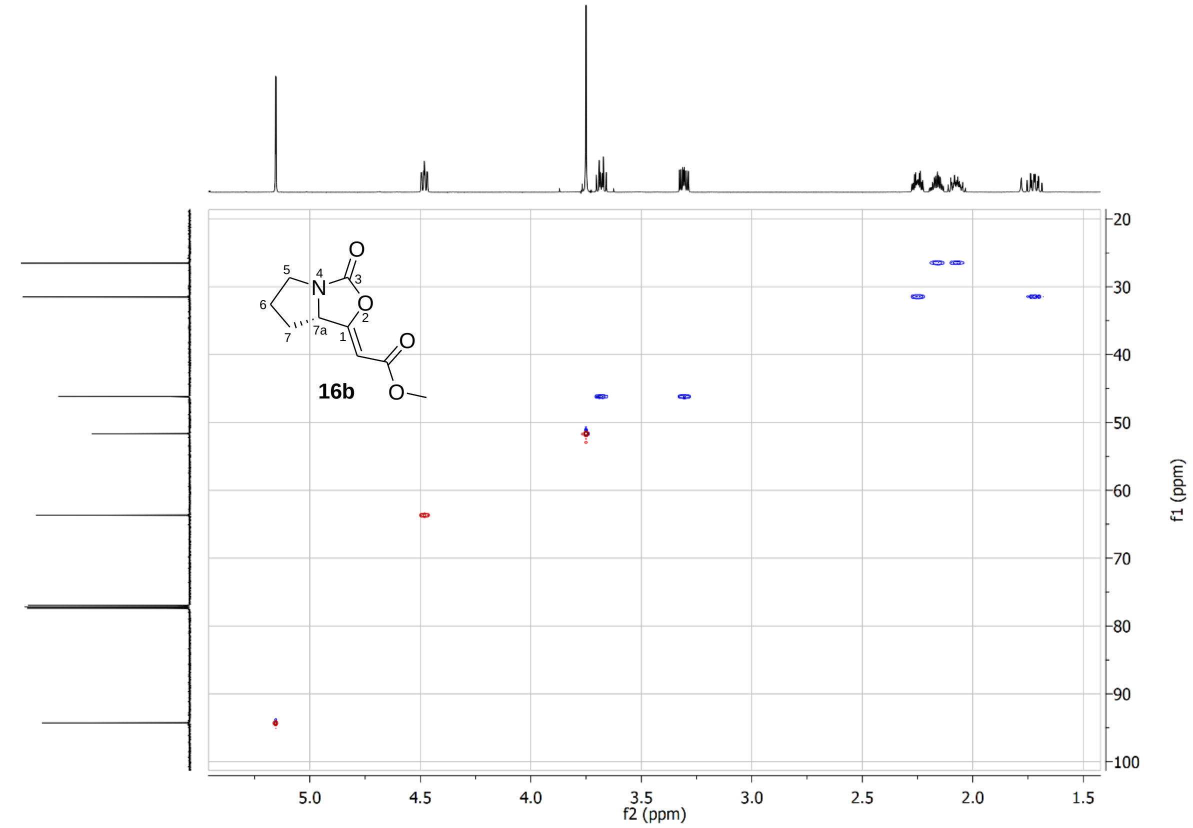
16b
f2 (ppm)
f1 (ppm)

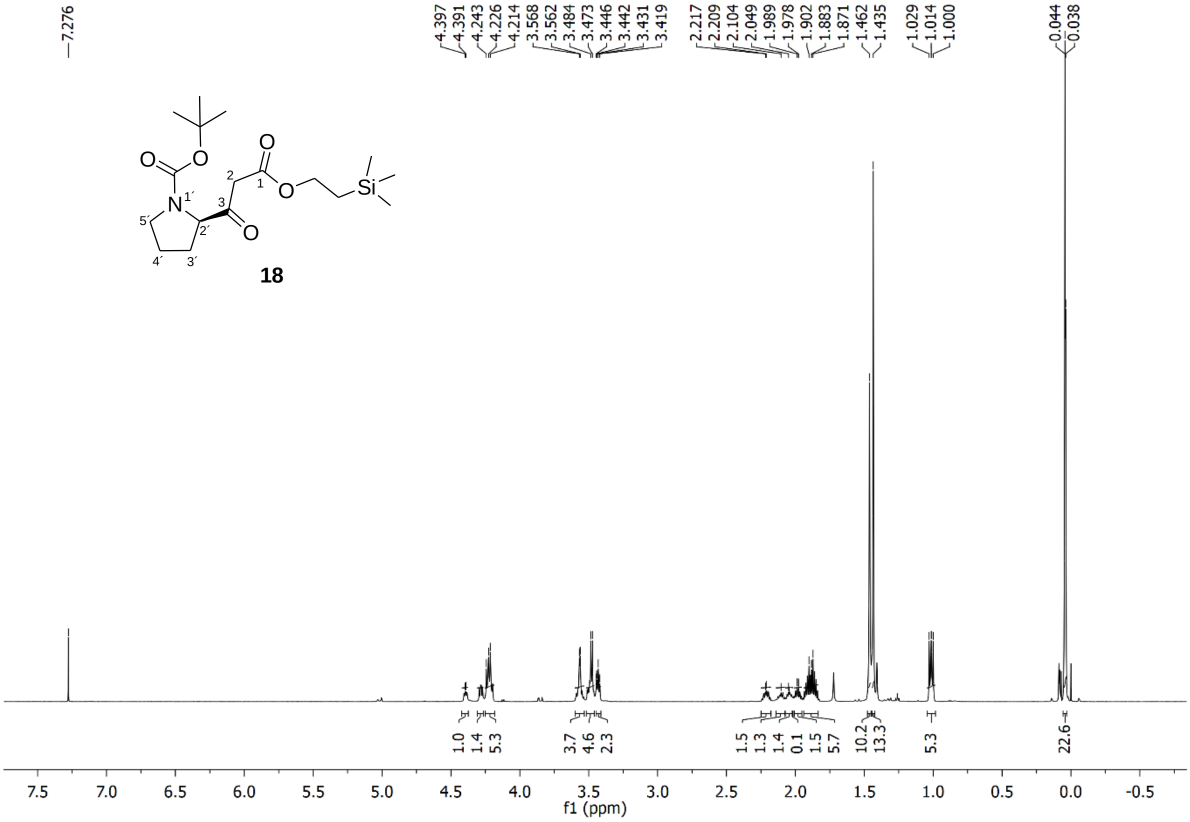
7.276
4.397
4.391
4.243
4.226
4.214
3.568
3.562
3.484
3.473
3.446
3.442
3.431
3.419
2.217
2.209
2.104
2.049
1.989
1.978
1.902
1.883
1.871
1.462
1.435
1.029
1.014
1.000
0.044
0.038
18
1.0
1.4
5.3
3.7
4.6
2.3
1.5
1.3
1.4
0.1
1.5
5.7
10.2
13.3
5.3
22.6
7.5 7.0 6.5 6.0 5.5 5.0 4.5 4.0 3.5 3.0 2.5 2.0 1.5 1.0 0.5 0.0 -0.5
f1 (ppm)

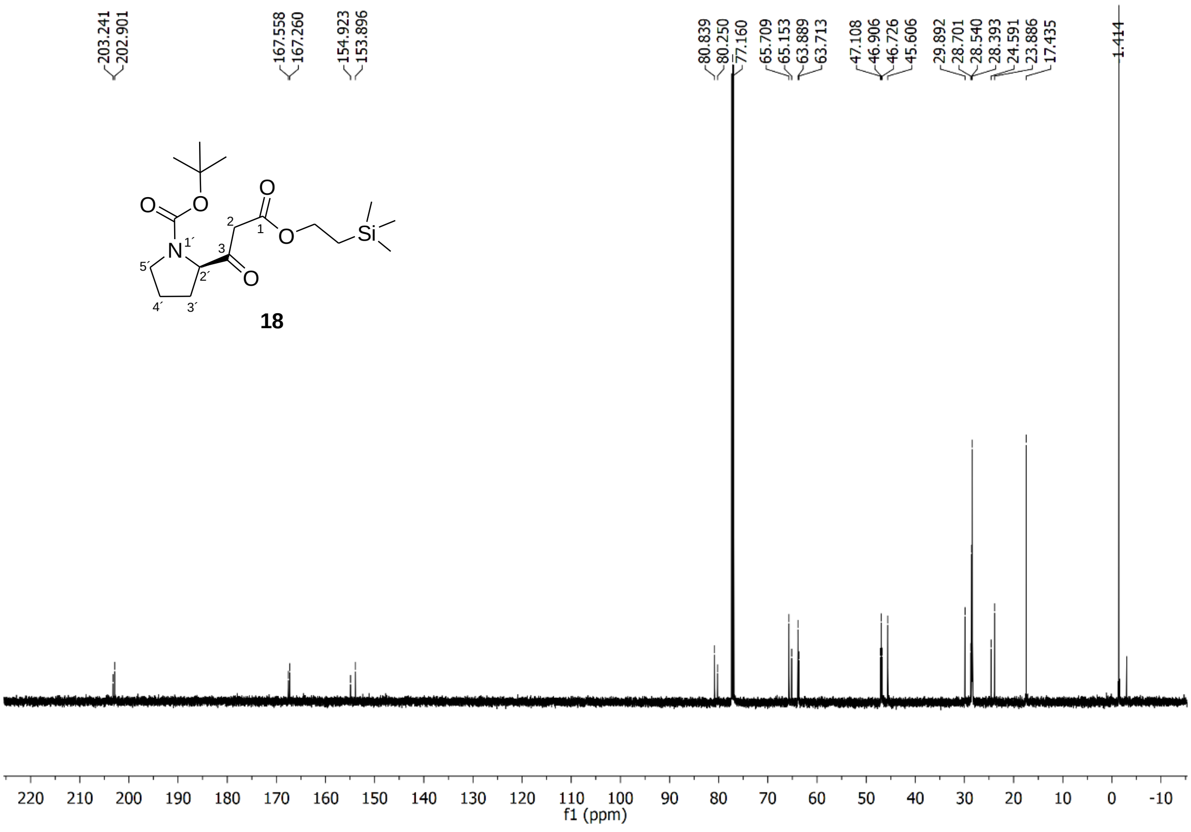
203.241
202.901
167.558
167.260
154.923
153.896
80.839
80.250
77.160
65.709
65.153
63.889
63.713
47.108
46.906
46.726
45.606
29.892
28.701
28.540
28.393
24.591
23.886
17.435
1.414
18
220 210 200 190 180 170 160 150 140 130 120 110 100 90 80 70 60 50 40 30 20 10 0 -10
f1 (ppm)

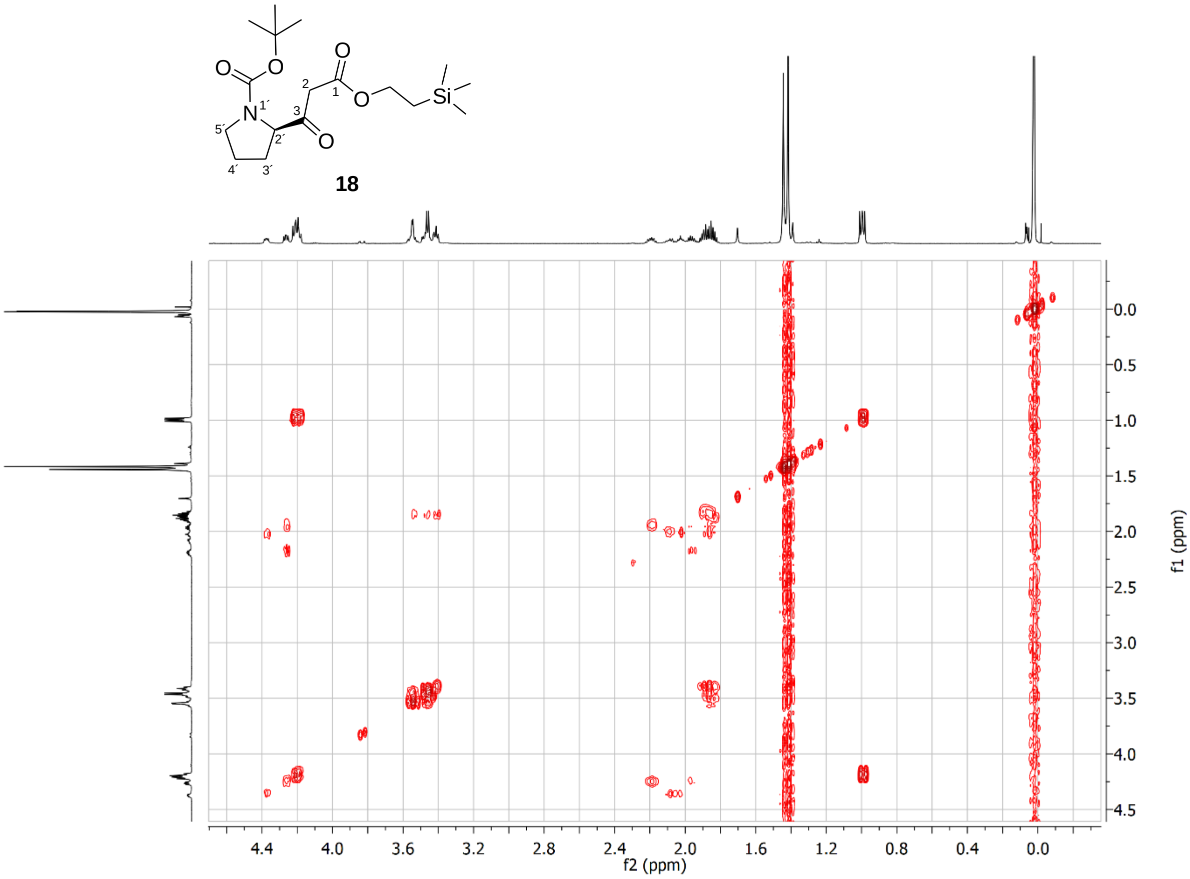
O
O
O
O
O
N
Si
1
2
3
1´
2´
3´
4´
5´
18
f2 (ppm)
f1 (ppm)

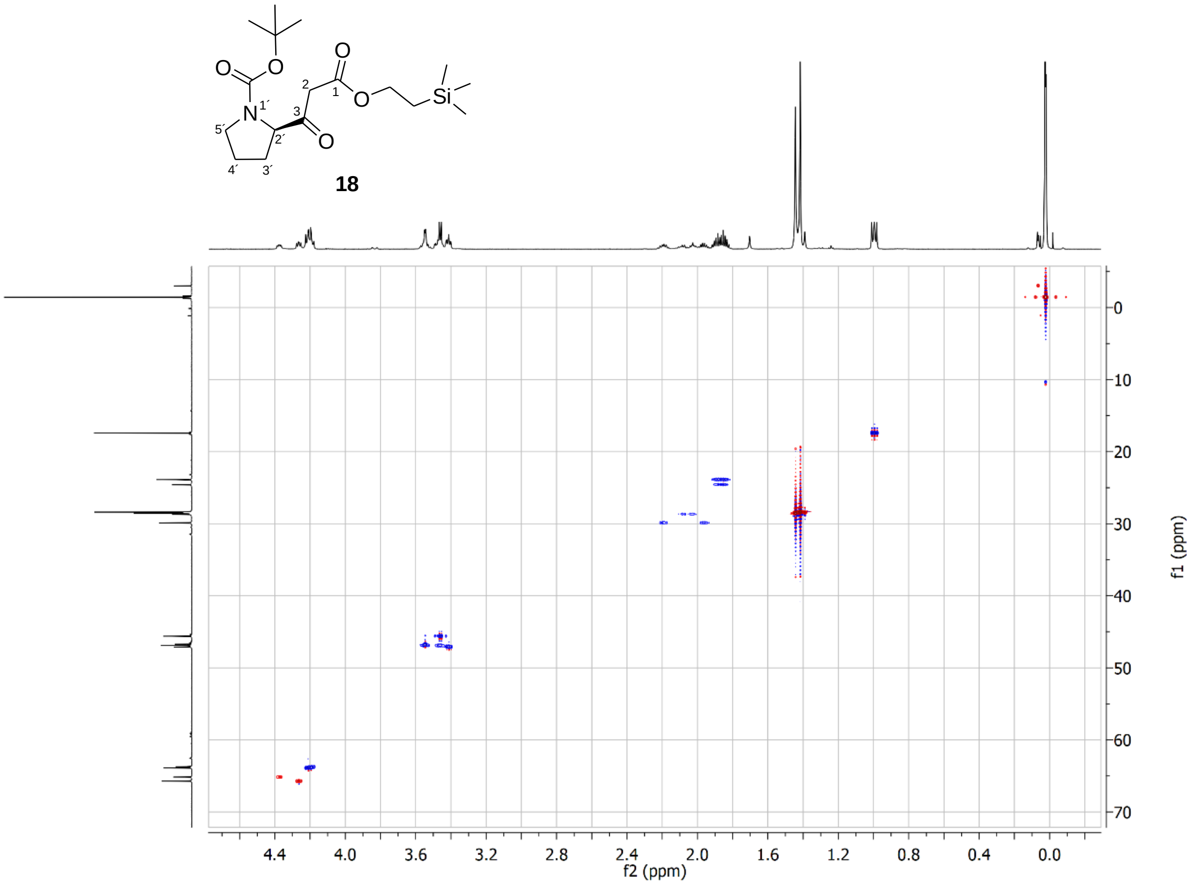
O
O
O
O
O
N
Si
1
2
3
1´
2´
3´
4´
5´
18
f2 (ppm)
f1 (ppm)

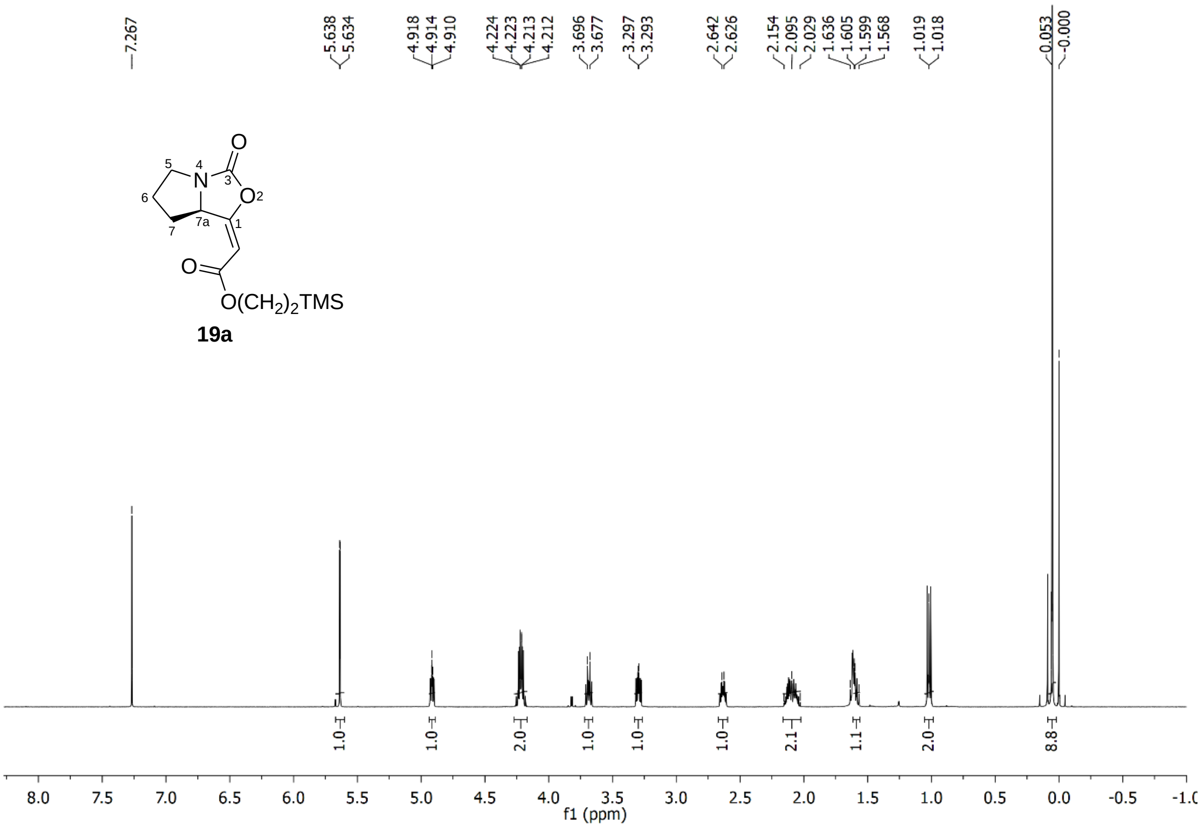
19a
f1 (ppm)

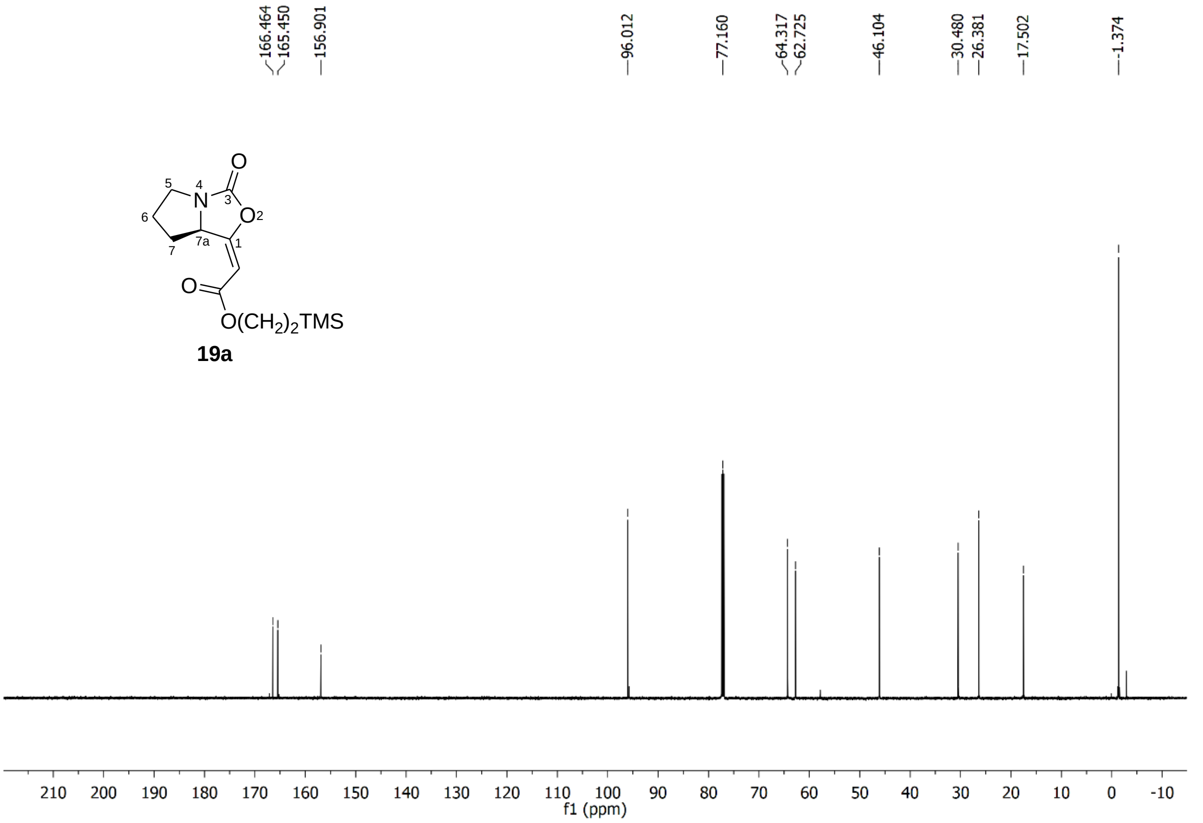
19a
f1 (ppm)

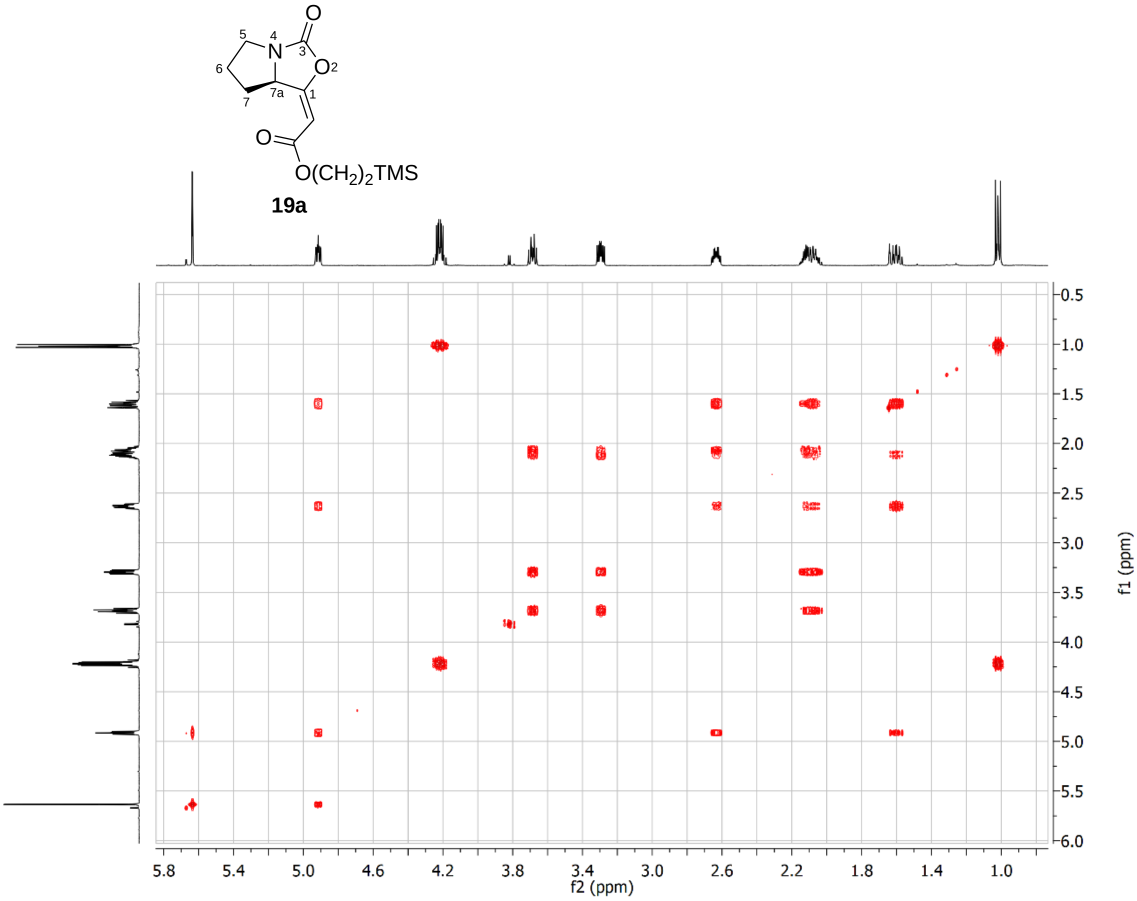

O(CH2)2TMS
19a
f2 (ppm)
f1 (ppm)

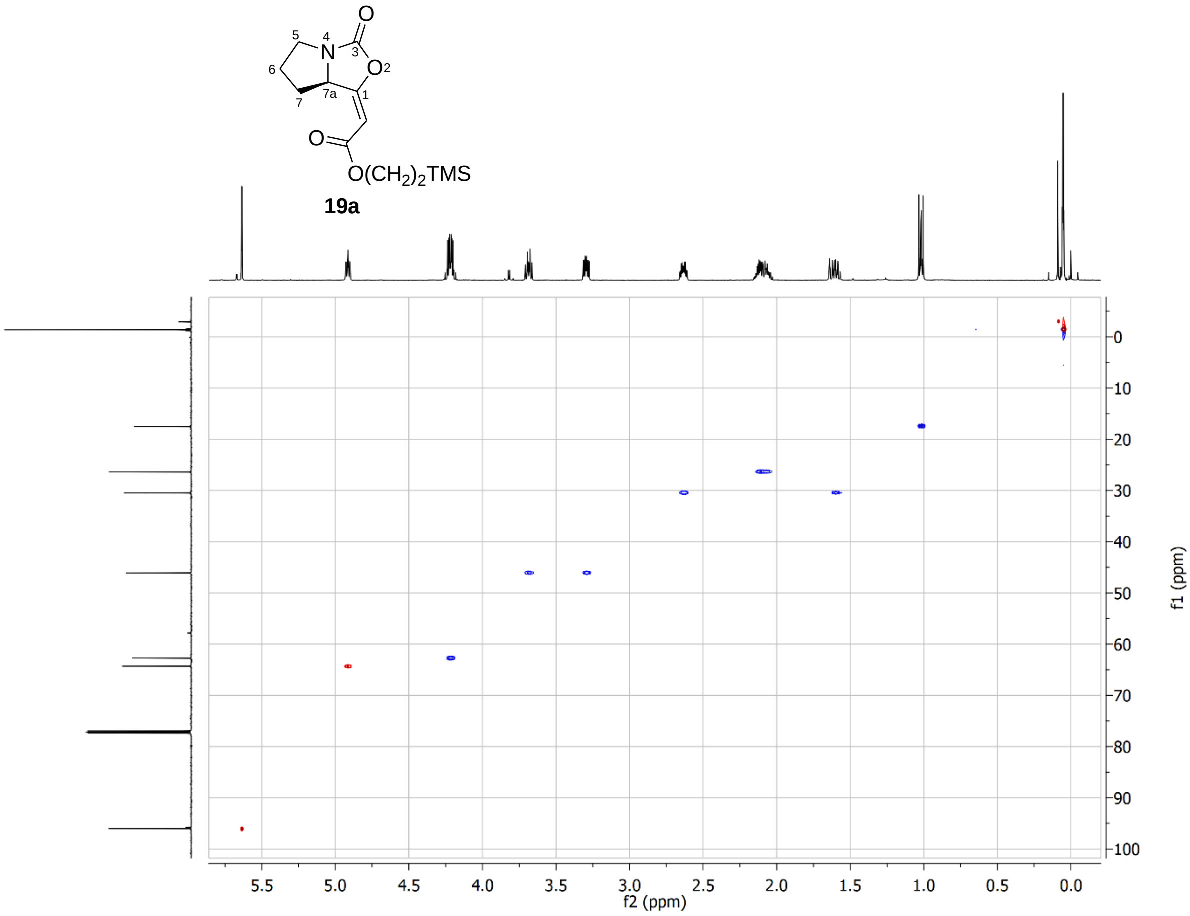

O(CH2)2TMS
19a
f2 (ppm)
f1 (ppm)

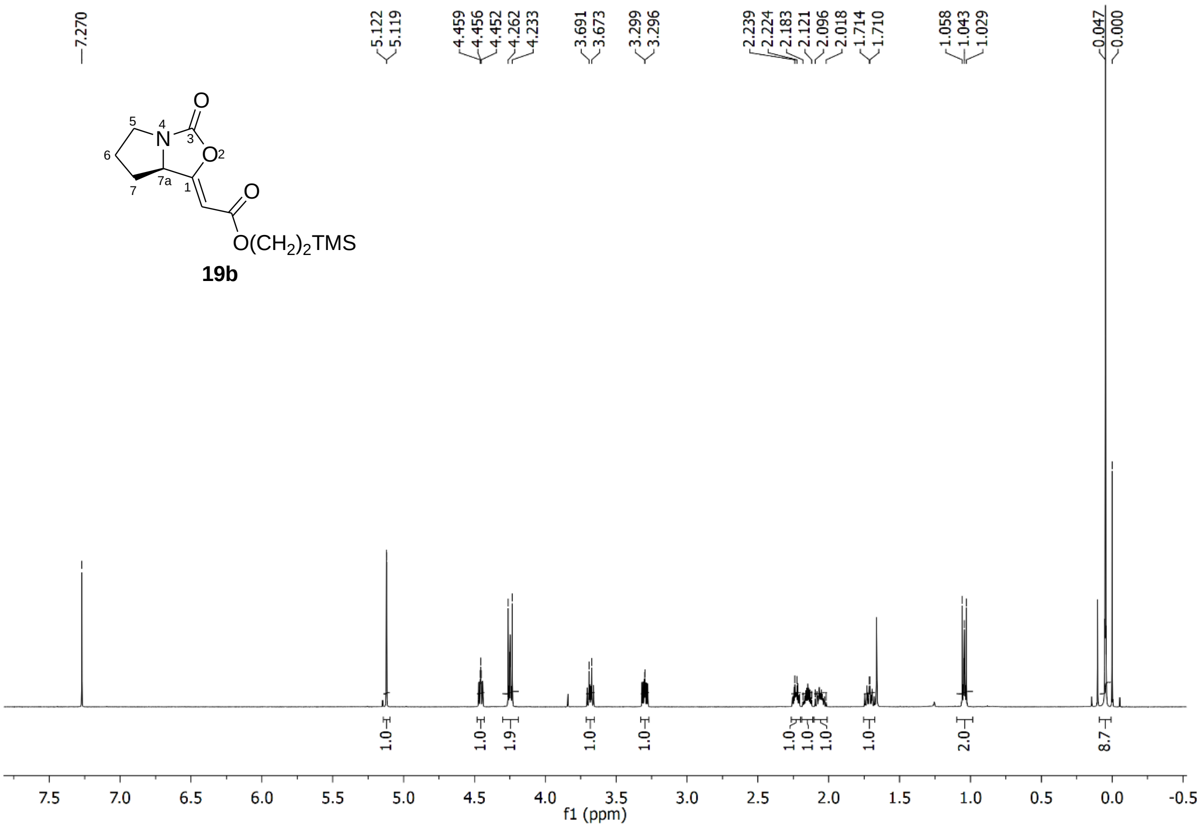
7.270
5.122
5.119
4.459
4.456
4.452
4.262
4.233
3.691
3.673
3.299
3.296
2.239
2.224
2.183
2.121
2.096
2.018
1.714
1.710
1.058
1.043
1.029
0.047
0.000
O
5
4
N
3
6
O
2
7
7a
1
O
O(CH2)2TMS
19b
1.0
1.0
1.9
1.0
1.0
1.0
1.0
1.0
1.0
2.0
8.7
7.5 7.0 6.5 6.0 5.5 5.0 4.5 4.0 3.5 3.0 2.5 2.0 1.5 1.0 0.5 0.0 -0.5
f1 (ppm)

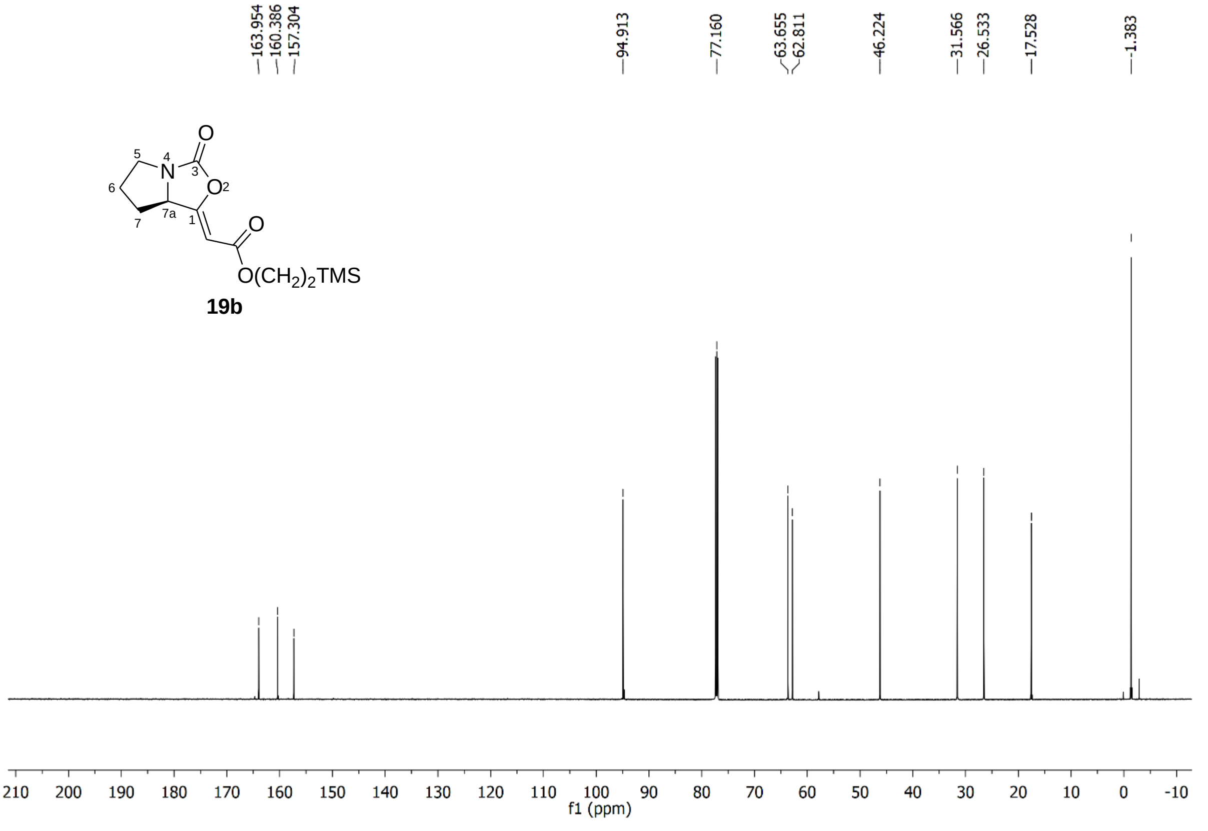
163.954
160.386
157.304
94.913
77.160
63.655
62.811
46.224
31.566
26.533
17.528
-1.383
O
5
4
N
3
6
O
2
7
7a
1
O
O(CH2)2TMS
19b
210 200 190 180 170 160 150 140 130 120 110 100 90 80 70 60 50 40 30 20 10 0 -10
f1 (ppm)

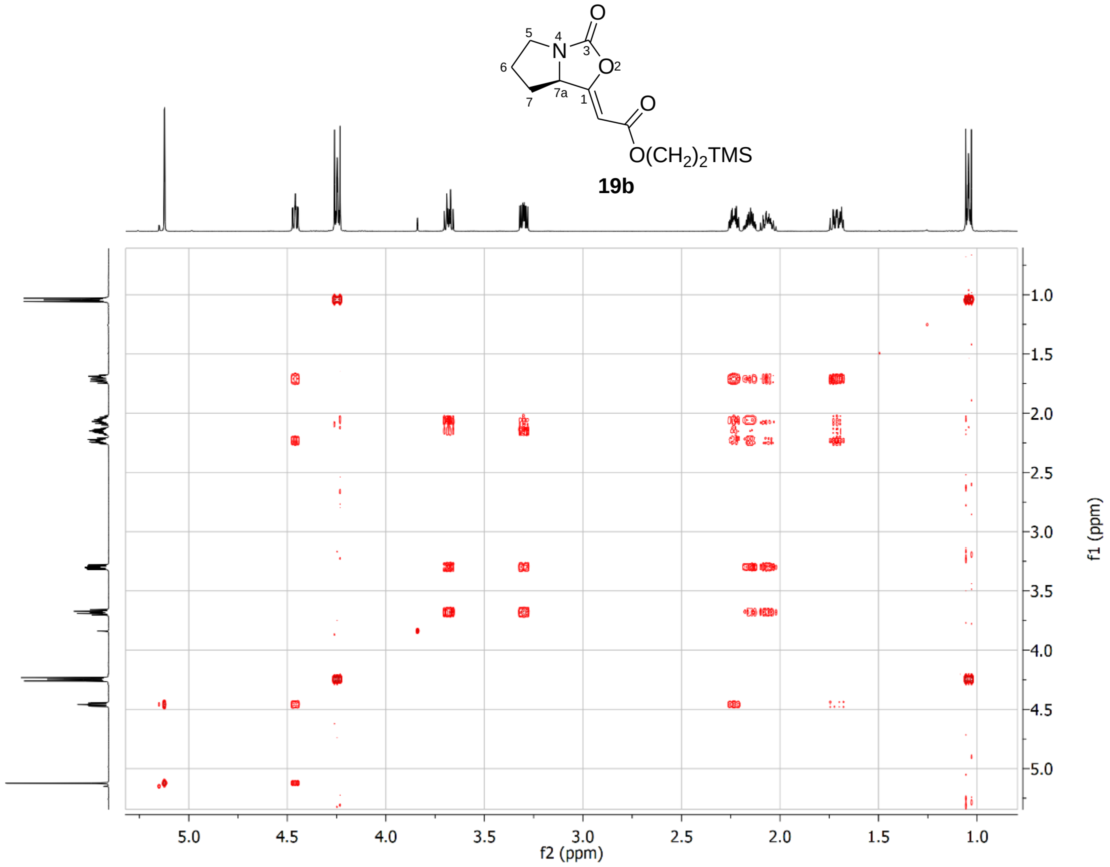
19b
O(CH2)2TMS
f2 (ppm)
f1 (ppm)

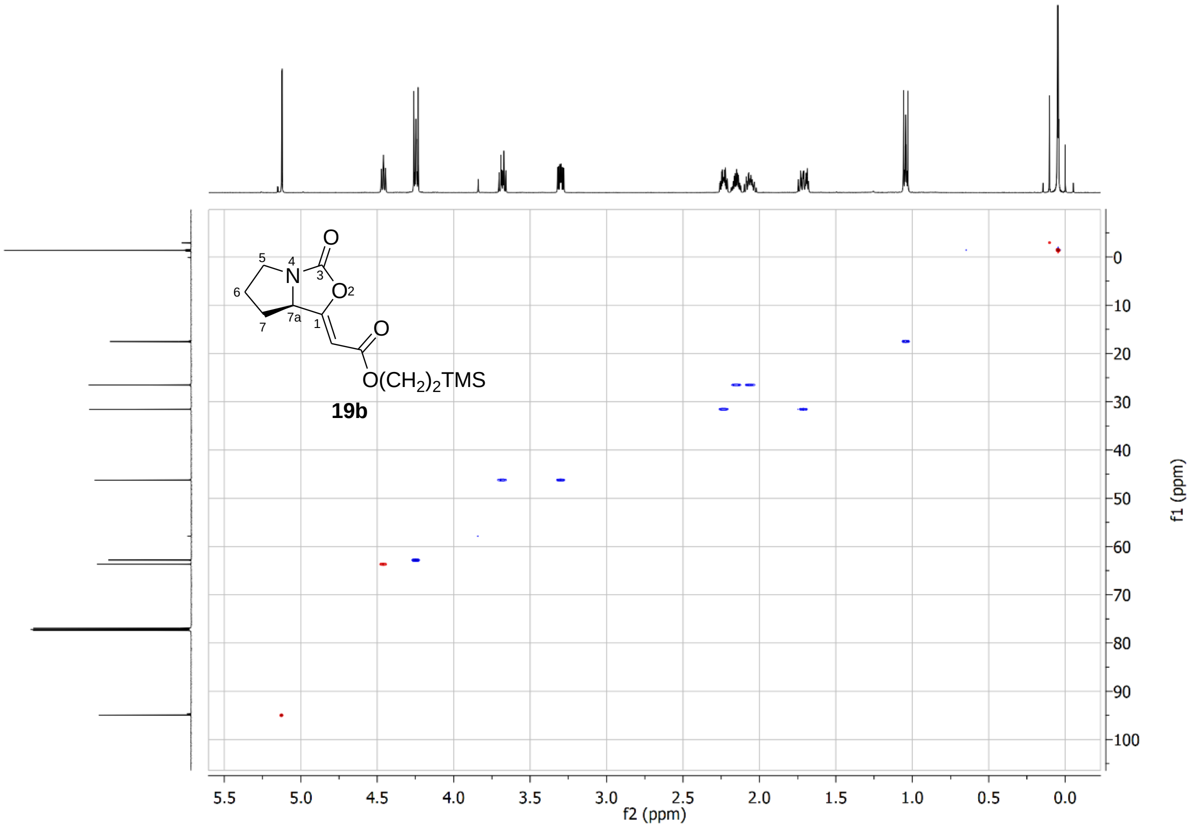
19b
O(CH2)2TMS
f2 (ppm)
f1 (ppm)

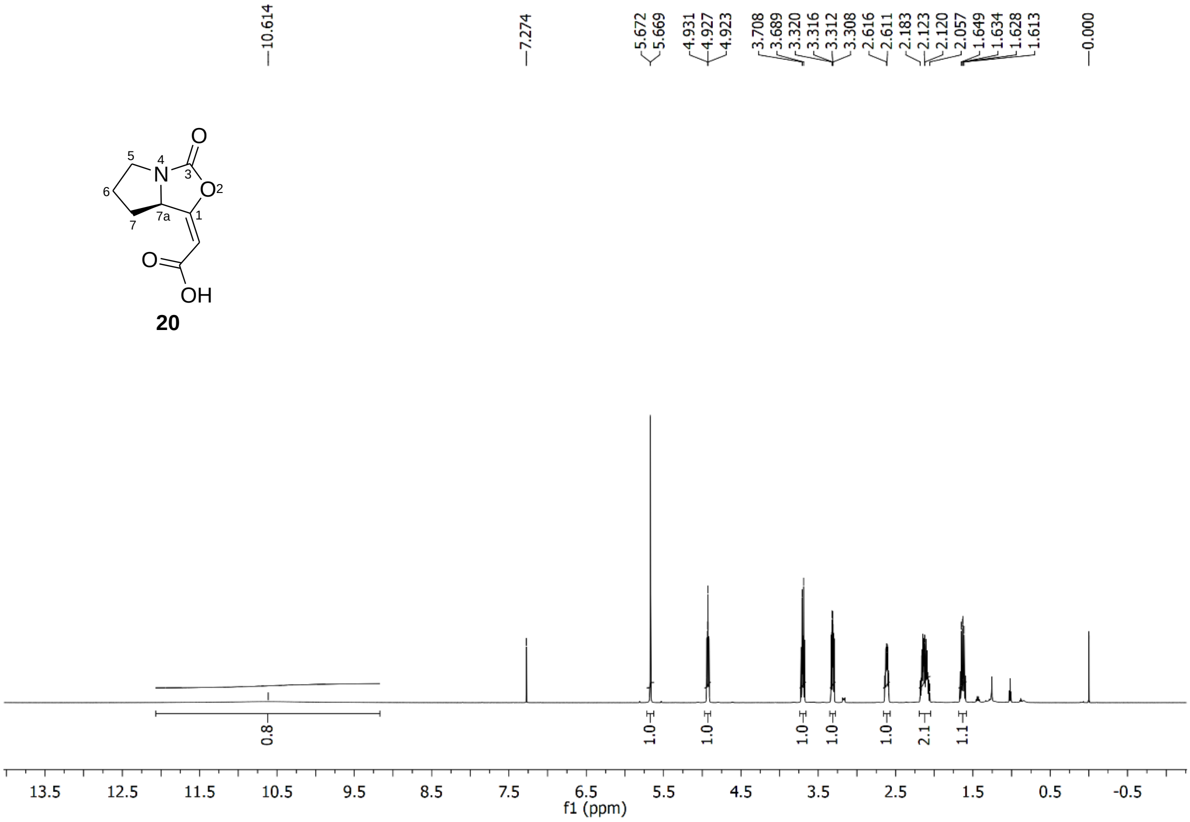
10.614
7.274
5.672
5.669
4.931
4.927
4.923
3.708
3.689
3.320
3.316
3.312
3.308
2.616
2.611
2.183
2.123
2.120
2.057
1.649
1.634
1.628
1.613
0.000
20
0.8
1.0
1.0
1.0
1.0
1.0
2.1
1.1
f1 (ppm)

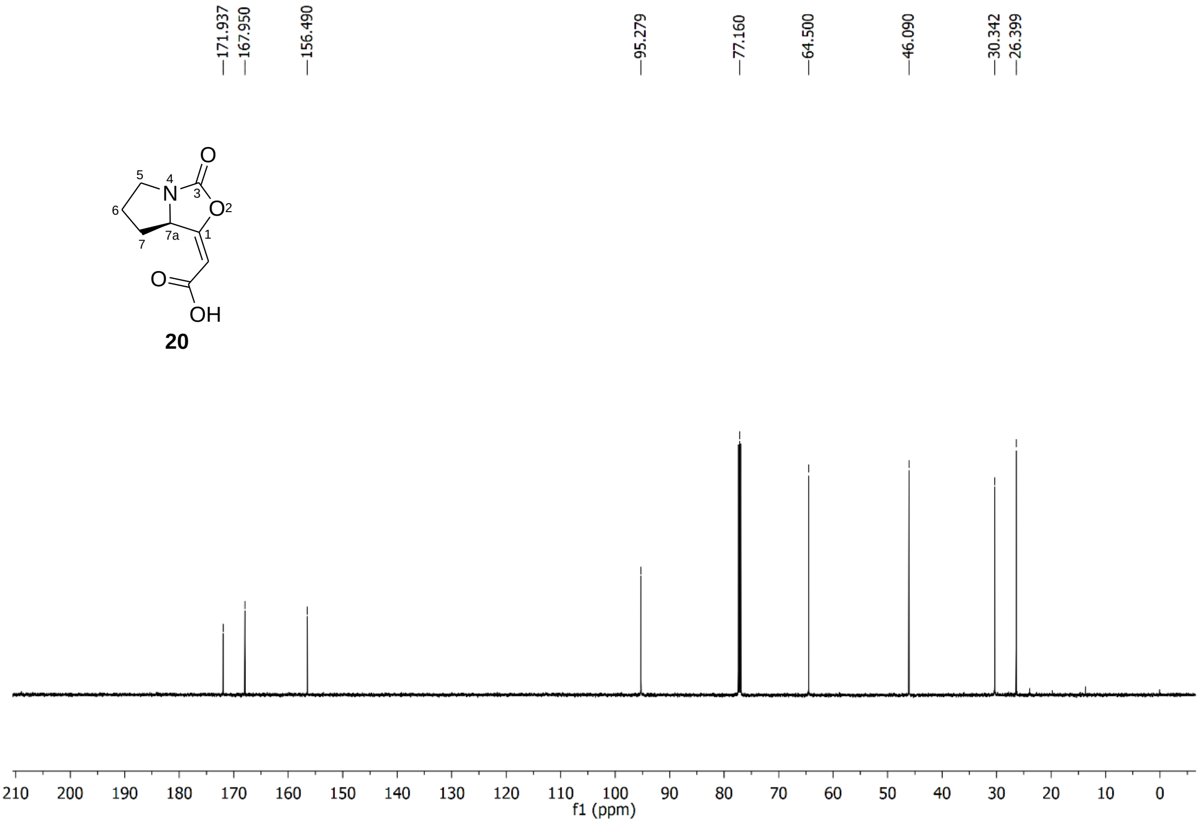
171.937
167.950
156.490
95.279
77.160
64.500
46.090
30.342
26.399
20
f1 (ppm)

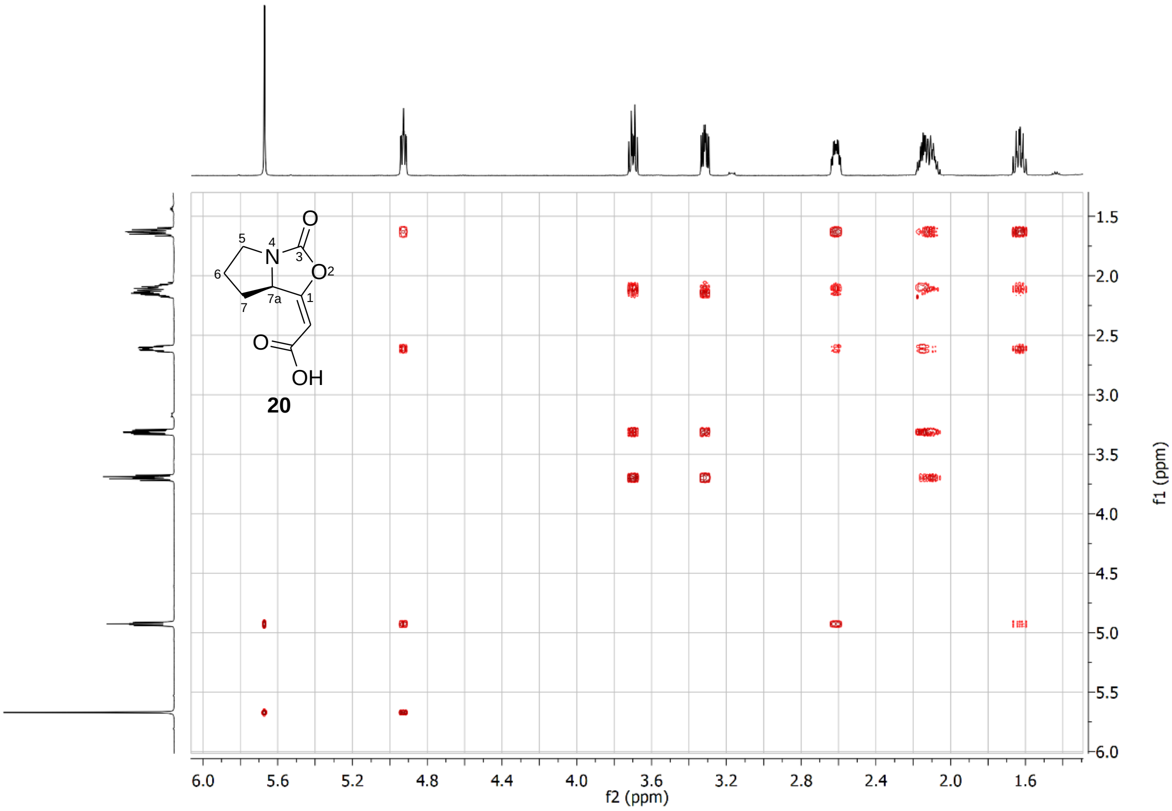

20
f2 (ppm)
f1 (ppm)

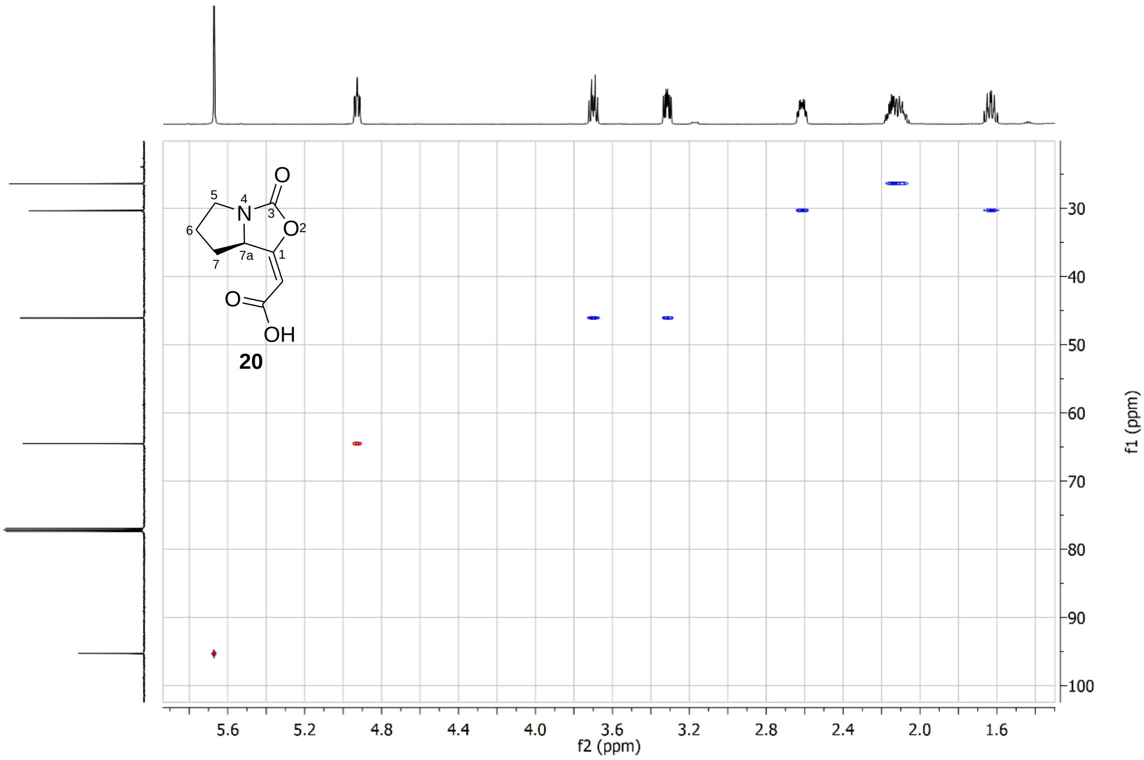

20
f2 (ppm)
f1 (ppm)

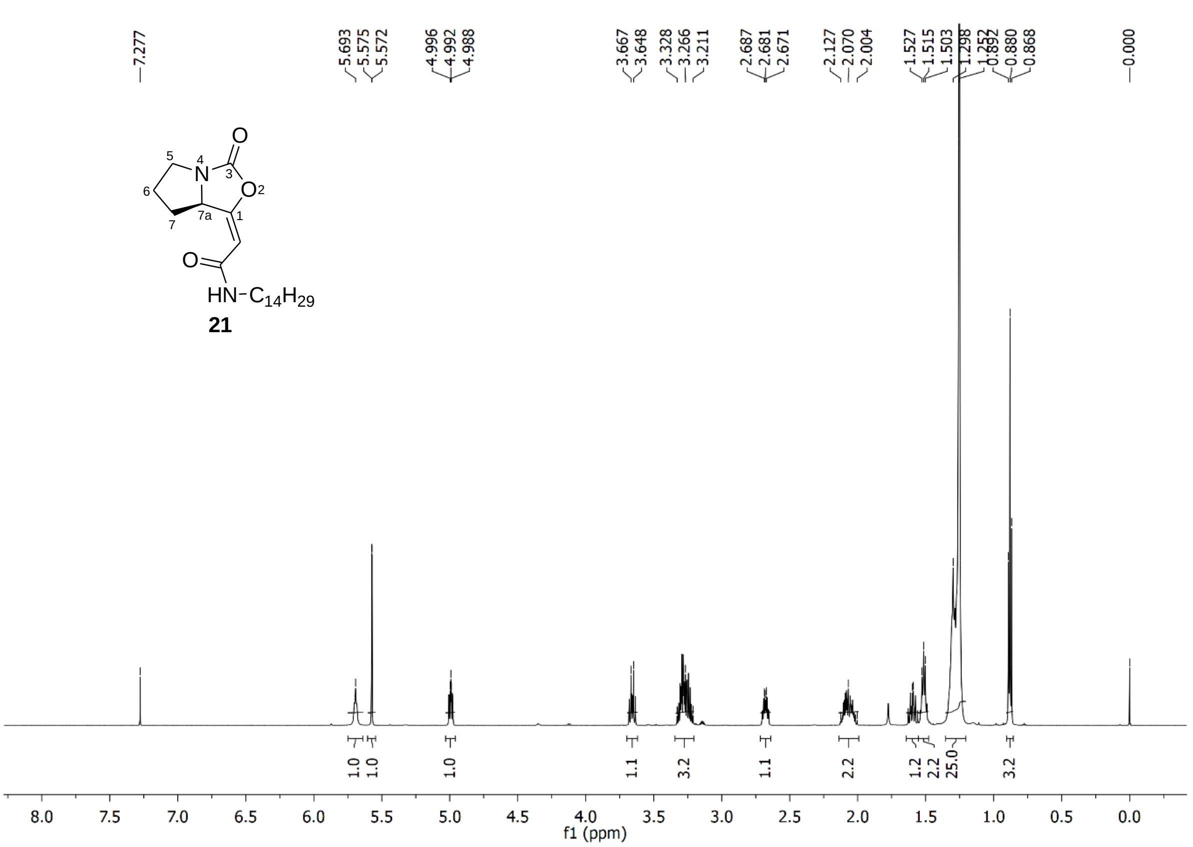
7.277
5.693
5.575
5.572
4.996
4.992
4.988
3.667
3.648
3.328
3.266
3.211
2.687
2.681
2.671
2.127
2.070
2.004
1.527
1.515
1.503
0.880
0.868
0.000
21
1.0
1.0
1.0
1.1
3.2
1.1
2.2
1.2
2.2
25.0
3.2
f1 (ppm)

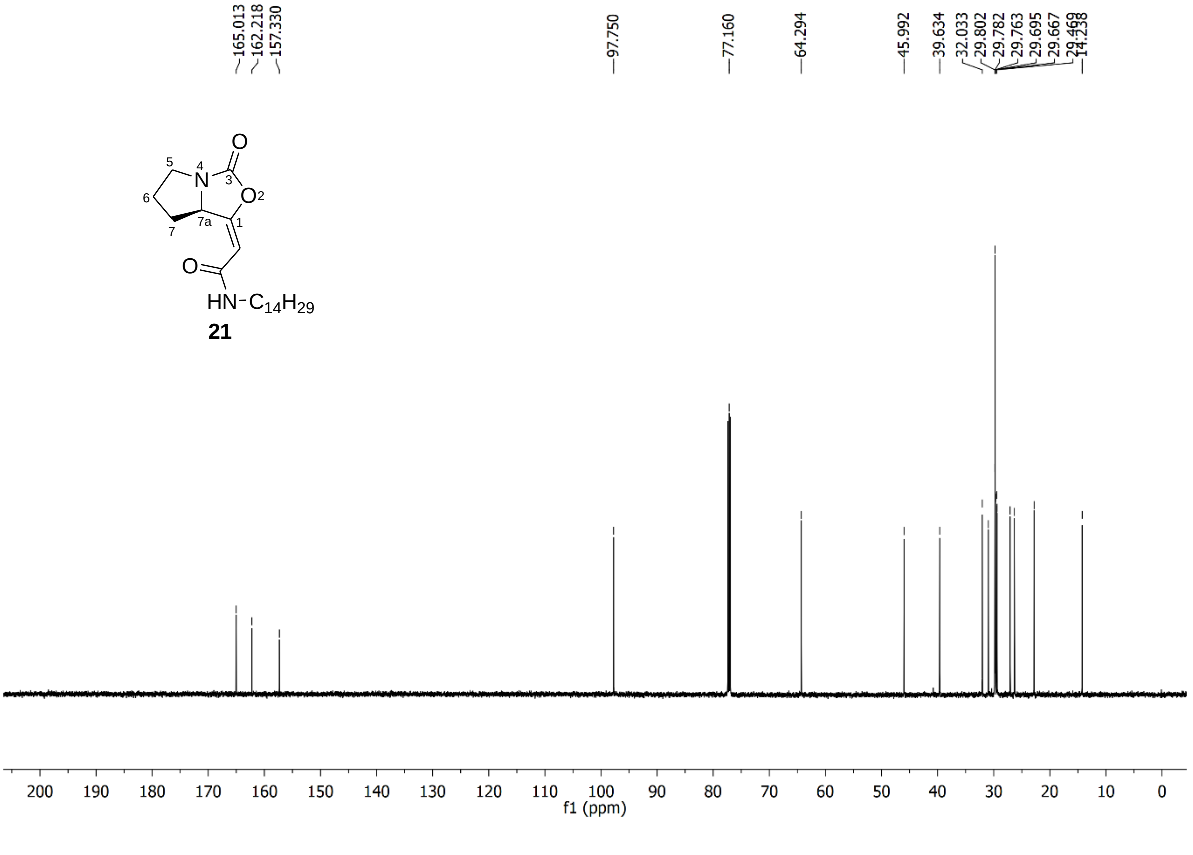
165.013
162.218
157.330
97.750
77.160
64.294
45.992
39.634
32.033
29.802
29.782
29.763
29.695
29.667
21
f1 (ppm)

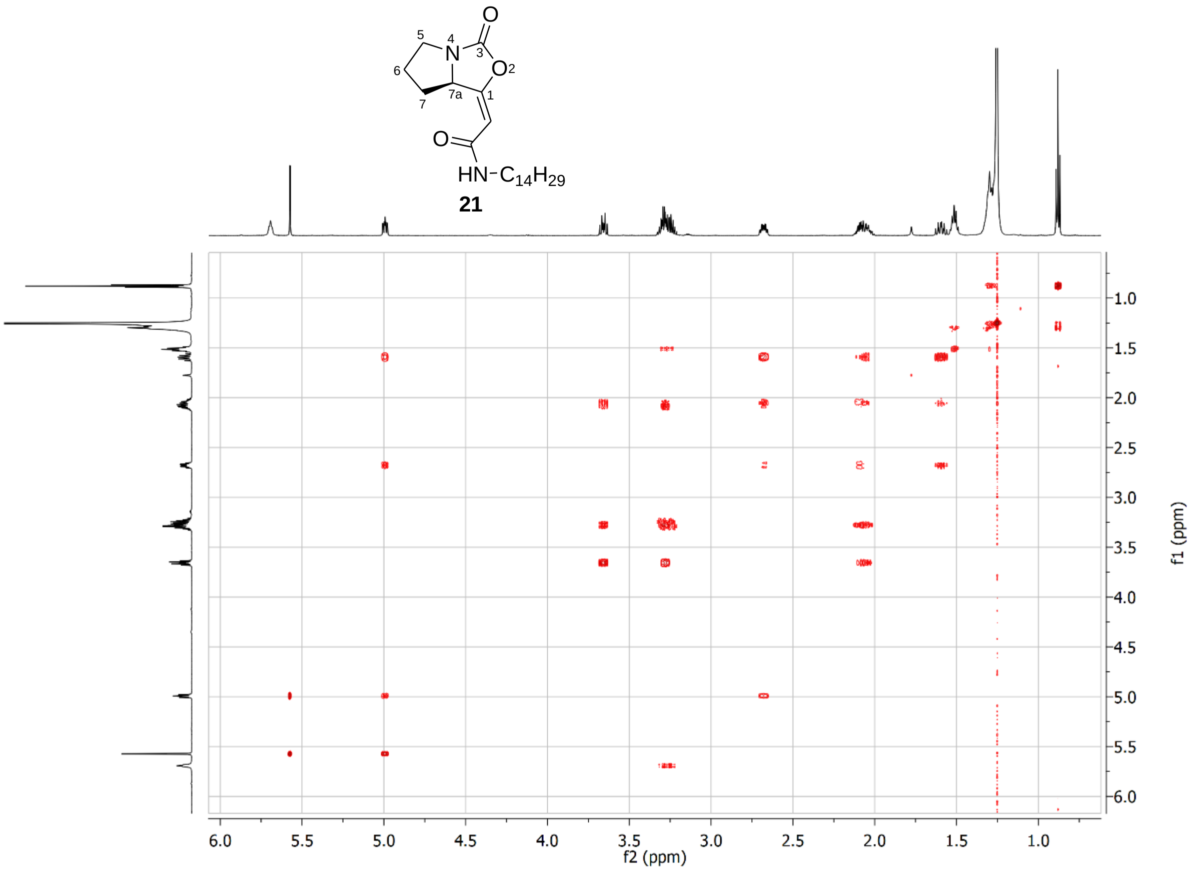
O
5
4
N
3
6
O2
7a
1
7
O
HN-C14H29
21
6.0 5.5 5.0 4.5 4.0 3.5 3.0 2.5 2.0 1.5 1.0
f2 (ppm)
1.0 1.5 2.0 2.5 3.0 3.5 4.0 4.5 5.0 5.5 6.0
f1 (ppm)

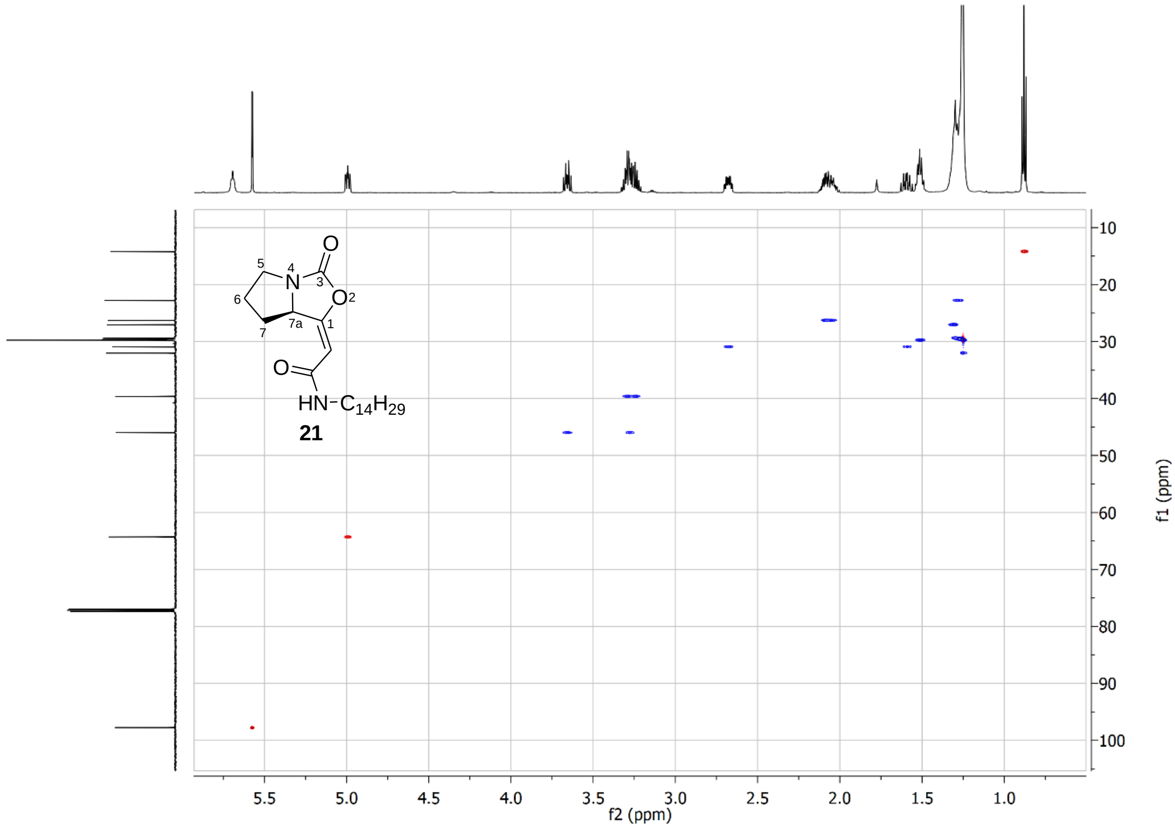
O
5
4
N
3
6
O2
7a
1
7
O
HN-C14H29
21
5.5 5.0 4.5 4.0 3.5 3.0 2.5 2.0 1.5 1.0
f2 (ppm)
10 20 30 40 50 60 70 80 90 100
f1 (ppm)

X-Ray Crystallography

Data collection and cell refinement of **16a** (Fig. X1), **20** (Fig. X2) and **21** (Fig. X3) were made by Stoe StadiVari diffractometer at 100 K using Pilatus3R 300K HPAD detector and microfocused source Xenocs Genix3D Cu HF (Cu Kα radiation λ = 1.54186 Å). The diffraction intensities were corrected for Lorentz and polarization factors. The structures were solved using SHELXT or SIR2011 programs[4,5] and refined by full-matrix least-squares procedure with SHELXL (version 2018/3).[6] The multi-scan absorption corrections were applied using Stoe LANA software.[7] Geometrical analyses were performed with SHELXL. The structures were drawn with OLEX2.[8] The absolute structure all compounds has been determined by Flack parameter using Parsons method, and by Hooft parameter.[9,10]

Crystal Data for $C_9H_{11}NO_4$ (**16a**) (M =197.19 g/mol): orthorhombic, space group $P2_12_12_1$ (no. 19), a = 7.2347(2) Å, b = 12.4282(2) Å, c = 20.6075(3) Å, V = 1852.91(7) $Å^3$, Z = 8, T = 100 K, μ(CuKα) = 0.952 mm^{-1},Dcalc = 1.414 g/cm^3, 64018 reflections measured (8.308° ≤ 2Θ ≤ 146.83°), 3678 unique (R_{int} = 0.0222, R_{sigma} = 0.0061) which were used in all calculations. The final R_1 was 0.0265 (I > 2σ(I)) and wR_2 was 0.0718 (all data). The Flack parameter x = 0.00(5). The Hooft parameter y = 0.005(9).

Crystal Data for $C_8H_9NO_4$ (**20**) (M =183.16 g/mol): monoclinic, space group $P2_1$ (no. 4), a = 9.5701(1) Å, b = 7.7138(2) Å, c = 11.0161(2) Å, β = 90.420(1)°, V = 813.21(3) $Å^3$, Z = 4, T = 100 K, μ(CuKα) = 1.040 mm^{-1}, Dcalc = 1.496 g/cm^3, 38003 reflections measured (8.026° ≤ 2Θ ≤ 143.004°), 3103 unique (R_{int} = 0.0168, R_{sigma} = 0.0071) which were used in all calculations. The final R_1 was 0.0223 (I>2.0σ(I)) and wR_2 was 0.0579 (all data). The Flack parameter x = 0.01(4). The Hooft parameter y = 0.005(18).

Crystal Data for $C_8H_9NO_4$ (**21**) (M =378.54 g/mol): monoclinic, space group $P2_1$ (no. 4), a = 9.5249(1) Å, b = 9.1755(1) Å, c = 24.7518(3) Å, β = 95.845(1)°, V = 2151.95(4) $Å^3$, Z = 4, T = 100 K, μ(CuKα) = 0.606 mm^{-1}, Dcalc = 1.168 g/cm^3, 68366 reflections measured (7.18° ≤ 2Θ ≤ 143.642°), 7376 unique (R_{int} = 0.0155, R_{sigma} = 0.0072) which were used in all calculations. The final R_1 was 0.0282 (I>2.0σ(I)) and wR_2 was 0.0806 (all data). The Flack parameter x = 0.00(9). The Hooft parameter y = 0.04(2).

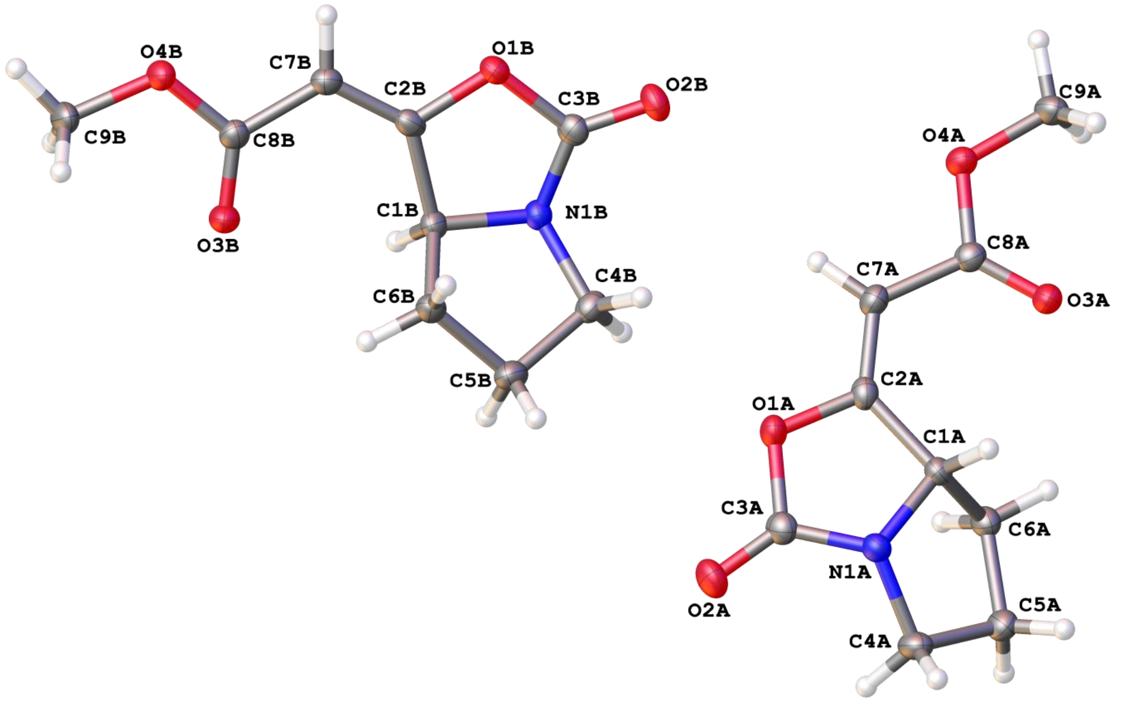

Fig. X1 Molecular structure of **16a** with the thermal ellipsoids shown at a 50% probability level.

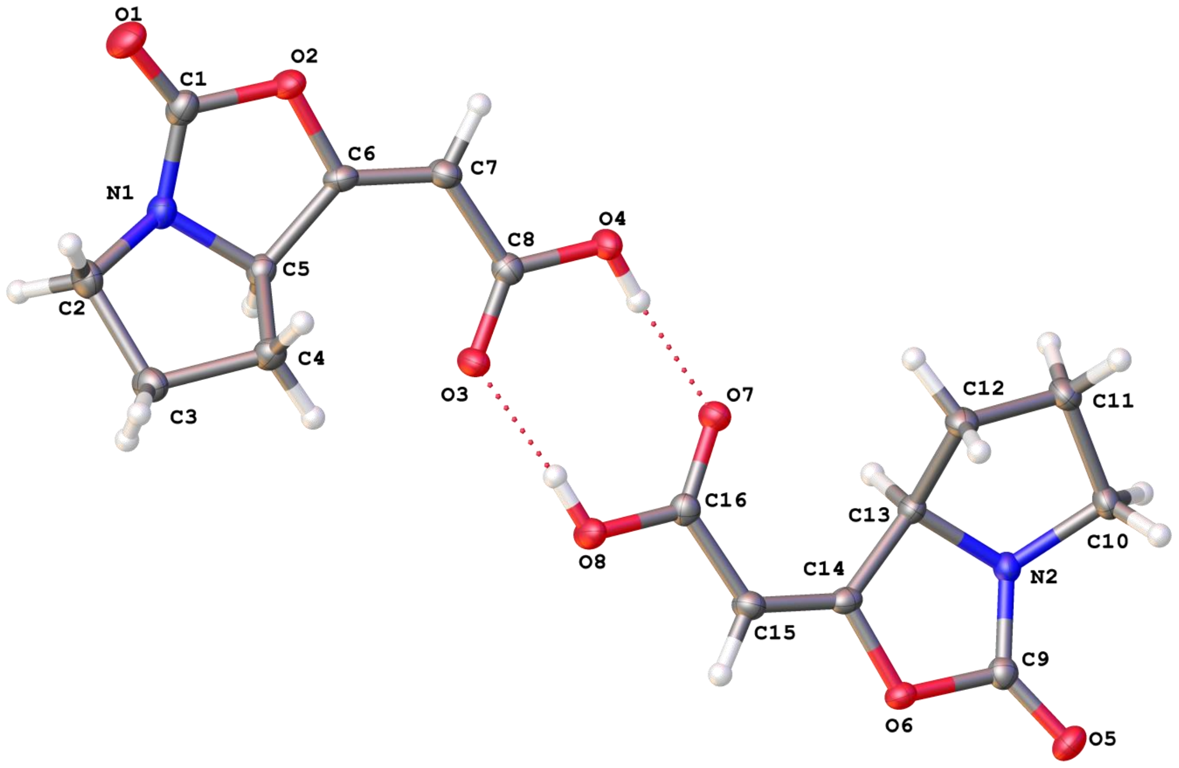

Fig. X2 Molecular structure of **20** with the thermal ellipsoids shown at a 50% probability level.

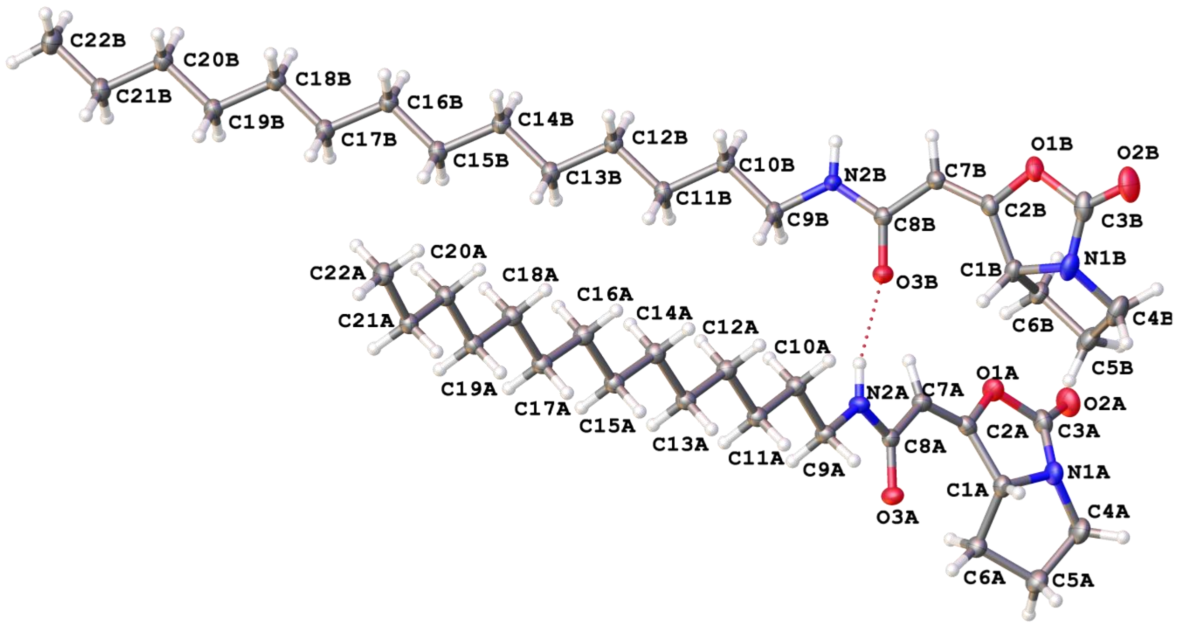

Fig. X3 Molecular structure of **21** with the thermal ellipsoids shown at a 50% probability level.

References and Notes:

(1) Dockerty, P.; Edens, J. G.; Tol, M. B.; Morales Angeles, D.; Domenech, A.; Liu, Y.; Hirsch, A. K. H.; Veening, J.-W.; Scheffers, D.-J.; Witte, M. D. *Org. Biomol. Chem.* **2017**, *15*, 894.

(2) Dichloromethane was used to furnish pure *E* isomer **19a**, then the eluent was changed to more polar mixture of CH_2Cl_2/EtOAc to furnish minor *Z* isomer **19b**.

(3) Dichloromethane was used to furnish impurities, then the eluent was changed to more polar mixture of CH_2Cl_2/MeOH/AcOH to furnish acid **20**.

(4) Sheldrick, G. M. *Acta Crystallogr., Sect. A* **2015**, *A71*, 3.

(5) Burla, M. C.; Camalli, R.; Camalli, M.; Carrozzini, B.; Cascarano, G. L.; Giacovazzo, C.; Mallamo, M.; Mazzone, A.; Polidori, G.; Spagna, R. *J. Appl. Crystallogr.* **2012**, *45*, 351.

(6) Sheldrick, G. M. *Acta Crystallogr., Sect. C* **2015**, *C71*, 3.

(7) Koziskova, J.; Hahn, F.; Richter, J.; Kozisek, J. *Acta Chim. Slovaca* **2016**, *9*, 136.

(8) Dolomanov, O. V.; Bourhis, L. J.; Gildea, R. J.; Howard, J. A. K.; Puschmann, H. *J. Appl. Crystallogr.* **2009**, *42*, 339.

(9) Parsons, S.; Flack, H. D.; Wagner, T. *Acta Crystallogr., Sect. B* **2013**, *B69*, 249.

(10) Hooft, R. W. W.; Straver, L. H.; Spek, A. L. *J. Appl. Crystallogr.* **2008**, *41*, 96.
